# Supplementary material for: Ferrocenyl Substituted Stannanethione and Stannaneselone
Source: Molecules. 2025 Jun 30;30(13):2826. doi: 10.3390/molecules30132826 (PMC12250751; doi:10.3390/molecules30132826)

checkCIF/PLATON report

Structure factors have been supplied for datablock(s) compound2, compound4a, compound4b, compound6, compound7, compound8

THIS REPORT IS FOR GUIDANCE ONLY. IF USED AS PART OF A REVIEW PROCEDURE FOR PUBLICATION, IT SHOULD NOT REPLACE THE EXPERTISE OF AN EXPERIENCED CRYSTALLOGRAPHIC REFEREE.

No syntax errors found. CIF dictionary Interpreting this report

Datablock: compound2

```
Bond precision:   C-C = 0.0049 A           Wavelength=0.71073

Cell:            a=19.3954(3)     b=35.2095(5)        c=21.6237(3)
                 alpha=90         beta=113.369(2)     gamma=90
Temperature:     100 K

                Calculated                     Reported
Volume          13555.5(4)                     13555.5(4)
Space group     P 21/n                         P 21/n
Hall group      -P 2yn                         -P 2yn
                C75.72 H97.15 Fe2 Se Sn,
Moiety formula  C76 H98 Fe2 Se Sn, C6 H6,      2(C76H98Fe2SeSn), C6H6
                0.282(C H3)
Sum formula     C158 H202 Fe4 Se2 Sn2          C158 H202 Fe4 Se2 Sn2
Mr              2719.95                        2719.88
Dx,g cm-3       1.333                          1.333
Z               4                              4
Mu (mm-1)       1.368                          1.368
F000            5688.0                         5688.0
F000'           5689.00
h,k,lmax        25,45,28                       25,45,28
Nref            31086                          31060
Tmin,Tmax       0.921,0.960                    0.243,1.000
Tmin'           0.872

Correction method= # Reported T Limits: Tmin=0.243 Tmax=1.000
AbsCorr = MULTI-SCAN

Data completeness= 0.999           Theta(max)= 27.485
```

R(reflections)= 0.0375(26324)

wR2(reflections)= 0.0962(31060)

S = 1.026

Npar= 1600

The following ALERTS were generated. Each ALERT has the format
test-name_ALERT_alert-type_alert-level.
Click on the hyperlinks for more details of the test.

Alert level B

```
PLAT250_ALERT_2_B Large U3/U1 Ratio for <U(i,j)> Tensor(Resd    3)        4.2 Note
```

Alert level C

```
PLAT042_ALERT_1_C Calc. and Reported MoietyFormula Strings  Differ     Please Check
              Calc: C75.72 H97.15 Fe2 Se Sn, C76 H98 Fe2 Se Sn, C6
                     H6, 0.282(C H3)
              Rep.: 2(C76H98Fe2SeSn), C6H6
PLAT213_ALERT_2_C Atom C154            has ADP max/min Ratio .....        3.7 prolat
PLAT220_ALERT_2_C NonSolvent   Resd 1  C   Ueq(max)/Ueq(min) Range        4.7 Ratio
PLAT220_ALERT_2_C NonSolvent   Resd 2  C   Ueq(max)/Ueq(min) Range        5.0 Ratio
PLAT222_ALERT_3_C NonSolvent Resd 1  H   Uiso(max)/Uiso(min) Range        5.7 Ratio
PLAT222_ALERT_3_C NonSolvent Resd 2  H   Uiso(max)/Uiso(min) Range        5.6 Ratio
PLAT260_ALERT_2_C Large Average Ueq of Residue Including      C162      0.297 Check
PLAT910_ALERT_3_C Missing FCF Reflection(s) Below Theta(Min)[Deg]=       1.66 Note
                1  1  0,   0  2  0,   1  2  0,  -1  0  1,  -1  1  1,   0  1  1,
                0  2  1,
PLAT911_ALERT_3_C Missing FCF Refl Between Thmin & STh/L=    0.600         12 Report
                3  2  0,   1  3  0,   2  3  0,   1  0  1,  -2  2  1,  -1  3  1,
                1  4  1,  -1  5  1,  -1  1  2,   1  2  2,  -1  3  2,   1  0  3,
PLAT971_ALERT_2_C Check Calcd Resid. Dens.  0.75Ang From Se1             1.81 eA-3
PLAT972_ALERT_2_C Check Calcd Resid. Dens.  0.82Ang From Se1            -1.57 eA-3
PLAT975_ALERT_2_C Check Calcd Resid. Dens.  0.67Ang From C56     .       0.67 eA-3
```

Alert level G

```
PLAT003_ALERT_2_G Number of Uiso or U(i,j)  Restrained non-H-Atoms         20 Report
PLAT083_ALERT_2_G SHELXL Second Parameter in WGHT  Unusually Large      22.58 Why ?
PLAT178_ALERT_4_G The CIF-Embedded .res File Contains SIMU Records          3 Report
PLAT188_ALERT_3_G A Non-default SIMU Restraint Value has been used     0.0010 Report
PLAT188_ALERT_3_G A Non-default SIMU Restraint Value has been used     0.0010 Report
PLAT188_ALERT_3_G A Non-default SIMU Restraint Value has been used     0.0010 Report
PLAT301_ALERT_3_G Main Residue  Disorder .............(Resd    1)         7% Note
PLAT301_ALERT_3_G Main Residue  Disorder .............(Resd    2)         4% Note
PLAT302_ALERT_4_G Anion/Solvent/Minor-Residue Disorder (Resd    4)       100% Note
PLAT304_ALERT_4_G Non-Integer Number of Atoms in ..... (Resd    1)     176.87 Check
PLAT304_ALERT_4_G Non-Integer Number of Atoms in ..... (Resd    4)       1.13 Check
PLAT343_ALERT_2_G Unusual sp?      Angle Range in Main Residue for         C1 Check
PLAT343_ALERT_2_G Unusual sp?      Angle Range in Main Residue for        C42 Check
PLAT343_ALERT_2_G Unusual sp?      Angle Range in Main Residue for        C78 Check
PLAT343_ALERT_2_G Unusual sp?      Angle Range in Main Residue for       C116 Check
PLAT367_ALERT_2_G Long?  C(sp?)-C(sp?) Bond  C49      - C53      .       1.54 Ang.
PLAT412_ALERT_2_G Short Intra XH3 .. XHn     H22      ..H211     .       2.03 Ang.
                                                       x,y,z  =     1_555 Check
PLAT412_ALERT_2_G Short Intra XH3 .. XHn     H34      ..H212     .       2.04 Ang.
```

```
                                                     x,y,z  =      1_555 Check
PLAT412_ALERT_2_G Short Intra XH3 .. XHn     H79      ..H221     .       2.04 Ang.
                                                     x,y,z  =      1_555 Check
PLAT412_ALERT_2_G Short Intra XH3 .. XHn     H85      ..H222     .       2.11 Ang.
                                                     x,y,z  =      1_555 Check
PLAT413_ALERT_2_G Short Inter XH3 .. XHn     H212     ..H26      .       1.97 Ang.
                                                     x,y,z  =      1_555 Check
PLAT413_ALERT_2_G Short Inter XH3 .. XHn     H100     ..H82      .       2.02 Ang.
                                       -1/2+x,1/2-y,-1/2+z  =      4_565 Check
PLAT432_ALERT_2_G Short Inter X...Y Contact  C49      ..C56      .       2.53 Ang.
                                                     x,y,z  =      1_555 Check
PLAT432_ALERT_2_G Short Inter X...Y Contact  C50      ..C56      .       2.88 Ang.
                                                     x,y,z  =      1_555 Check
PLAT432_ALERT_2_G Short Inter X...Y Contact  C53      ..C56      .       1.77 Ang.
                                                     x,y,z  =      1_555 Check
PLAT432_ALERT_2_G Short Inter X...Y Contact  C163     ..C56      .       3.04 Ang.
                                                     x,y,z  =      1_555 Check
PLAT432_ALERT_2_G Short Inter X...Y Contact  C165     ..C56      .       2.83 Ang.
                                                     x,y,z  =      1_555 Check
PLAT773_ALERT_2_G Check long C-C Bond in CIF: C35      --C38             1.71 Ang.
PLAT773_ALERT_2_G Check long C-C Bond in CIF: C53      --C56             1.77 Ang.
PLAT794_ALERT_5_G Tentative Bond Valency for Fe1       (II)      .       2.19 Info
PLAT794_ALERT_5_G Tentative Bond Valency for Fe2       (II)      .       2.17 Info
PLAT794_ALERT_5_G Tentative Bond Valency for Fe3       (II)      .       2.19 Info
PLAT794_ALERT_5_G Tentative Bond Valency for Fe4       (II)      .       2.13 Info
PLAT860_ALERT_3_G Number of Least-Squares Restraints ............        144 Note
PLAT883_ALERT_1_G Absent Datum for _atom_sites_solution_primary ..     Please Do !
PLAT912_ALERT_4_G Missing # of FCF Reflections Above STh/L=  0.600         5 Note
PLAT933_ALERT_2_G Number of HKL-OMIT Records in Embedded .res File        13 Note
               -1  1  2,   1  2  0,  -1  3  2,   3  2  0,   1  2  2,   1  4  1,
               -2  2  1,  -1  3  1,   1  3  0,   1  0  1,  -1  5  1,   1  0  3,
                2  3  0,
PLAT960_ALERT_3_G Number of Intensities with I < - 2*Sigma(I) ....         5 Check
PLAT965_ALERT_2_G The SHELXL WEIGHT Optimisation has not Converged     Please Check
PLAT967_ALERT_5_G Note: Two-Theta Cutoff Value in Embedded .res ..       55.0 Degree
PLAT969_ALERT_5_G The 'Henn et al.' R-Factor-gap value ..........      4.223 Note
              Predicted wR2: Based on SigI**2  2.28 or SHELX Weight  9.37
PLAT978_ALERT_2_G Number C-C Bonds with Positive Residual Density.         1 Info
```

```
   0 ALERT level A = Most likely a serious problem - resolve or explain
   1 ALERT level B = A potentially serious problem, consider carefully
  12 ALERT level C = Check. Ensure it is not caused by an omission or oversight
  42 ALERT level G = General information/check it is not something unexpected

   2 ALERT type 1 CIF construction/syntax error, inconsistent or missing data
  31 ALERT type 2 Indicator that the structure model may be wrong or deficient
  11 ALERT type 3 Indicator that the structure quality may be low
   5 ALERT type 4 Improvement, methodology, query or suggestion
   6 ALERT type 5 Informative message, check
```

Datablock: compound4a

Bond precision: C-C = 0.0031 A   Wavelength=0.71073

Cell:	a=15.4833(2)	b=22.5112(2)	c=20.9383(3)
	alpha=90	beta=108.018(1)	gamma=90
Temperature:	103 K		

	Calculated	Reported
Volume	6940.10(15)	6940.10(15)
Space group	P 21/n	P 21/n
Hall group	-P 2yn	-P 2yn
Moiety formula	C76 H98 Fe2 S4 Sn	C76 H98 Fe2 S4 Sn
Sum formula	C76 H98 Fe2 S4 Sn	C76 H98 Fe2 S4 Sn
Mr	1370.20	1370.17
Dx,g cm-3	1.311	1.311
Z	4	4
Mu (mm-1)	0.931	0.931
F000	2880.0	2880.0
F000'	2882.83	
h,k,lmax	20,30,28	20,30,28
Nref	17882	17073
Tmin,Tmax	0.915,0.963	0.913,0.964
Tmin'	0.911	

Correction method= # Reported T Limits: Tmin=0.913 Tmax=0.964
AbsCorr = MULTI-SCAN

Data completeness= 0.955 Theta(max)= 28.660

R(reflections)= 0.0333(15423) wR2(reflections)= 0.0765(17073)

S = 1.069 Npar= 772

The following ALERTS were generated. Each ALERT has the format
test-name_ALERT_alert-type_alert-level.
Click on the hyperlinks for more details of the test.

Alert level C

```
PLAT094_ALERT_2_C Ratio of Maximum / Minimum Residual Density ....       3.16 Report
PLAT906_ALERT_3_C Large K Value in the Analysis of Variance ......      2.513 Check
PLAT911_ALERT_3_C Missing FCF Refl Between Thmin & STh/L=    0.600          6 Report
                6   2   0,    2   8   2,   -6   9   3,   -5   0   7,   -5   6   8,   -3   0  15,
PLAT971_ALERT_2_C Check Calcd Resid. Dens.  0.92Ang From S2              1.73 eA-3
```

Alert level G

```
PLAT066_ALERT_1_G Predicted and Reported Tmin&Tmax Range Identical          ? Check
PLAT083_ALERT_2_G SHELXL Second Parameter in WGHT  Unusually Large       9.01 Why ?
PLAT142_ALERT_4_G s.u. on b - Axis Small or Missing .............    0.00020 Ang.
```

```
PLAT232_ALERT_2_G Hirshfeld Test Diff (M-X)  Sn1      --S1       .        7.5 s.u.
PLAT343_ALERT_2_G Unusual sp?      Angle Range in Main Residue for         C1 Check
PLAT343_ALERT_2_G Unusual sp?      Angle Range in Main Residue for        C39 Check
PLAT794_ALERT_5_G Tentative Bond Valency for Fe1       (II)      .       2.05 Info
PLAT794_ALERT_5_G Tentative Bond Valency for Fe2       (II)      .       2.05 Info
PLAT883_ALERT_1_G Absent Datum for _atom_sites_solution_primary ..     Please Do !
PLAT899_ALERT_4_G SHELXL2018 is Outdated   and Succeeded by SHELXL     2019/3 Note
PLAT910_ALERT_3_G Missing FCF Reflection(s) Below Theta(Min)[Deg]=       1.65 Note
               -1  0  1,   0  1  1,
PLAT912_ALERT_4_G Missing # of FCF Reflections Above STh/L=  0.600        801 Note
PLAT933_ALERT_2_G Number of HKL-OMIT Records in Embedded .res File          9 Note
               -3  0 15,   8  9 19,  -7 24 16,  -6  9  3,  -9 24 15,  -5  6  8,
               -5  0  7,   6  2  0,   2  8  2,
PLAT965_ALERT_2_G The SHELXL WEIGHT Optimisation has not Converged     Please Check
PLAT969_ALERT_5_G The 'Henn et al.' R-Factor-gap value ..........      5.377 Note
              Predicted wR2: Based on SigI**2  1.42 or SHELX Weight  7.15
PLAT978_ALERT_2_G Number C-C Bonds with Positive Residual Density.         14 Info
```

```
   0 ALERT level A = Most likely a serious problem - resolve or explain
   0 ALERT level B = A potentially serious problem, consider carefully
   4 ALERT level C = Check. Ensure it is not caused by an omission or oversight
  16 ALERT level G = General information/check it is not something unexpected

   2 ALERT type 1 CIF construction/syntax error, inconsistent or missing data
   9 ALERT type 2 Indicator that the structure model may be wrong or deficient
   3 ALERT type 3 Indicator that the structure quality may be low
   3 ALERT type 4 Improvement, methodology, query or suggestion
   3 ALERT type 5 Informative message, check
```

Datablock: compound4b

```
Bond precision:    C-C = 0.0045 A            Wavelength=0.71073

Cell:               a=18.3966(2)      b=30.5224(4)      c=13.0978(2)
                    alpha=90          beta=90           gamma=90
Temperature:        103 K
```

	Calculated	Reported
Volume	7354.53(17)	7354.53(17)
Space group	P n n a	P n n a
Hall group	-P 2a 2bc	-P 2a 2bc
Moiety formula	C76 H98 Fe2 S5.20 Sn	?
Sum formula	C76 H98 Fe2 S5.20 Sn	C76 H98 Fe2 S5.20 Sn
Mr	1408.67	1408.64
Dx,g cm-3	1.272	1.272
Z	4	4
Mu (mm-1)	0.913	0.913
F000	2956.8	2957.0
F000'	2960.23	
h,k,lmax	24,41,17	24,41,17
Nref	9520	9273
Tmin,Tmax	0.916,0.955	0.914,0.956
Tmin'	0.913	

Correction method= # Reported T Limits: Tmin=0.914 Tmax=0.956
AbsCorr = MULTI-SCAN

Data completeness= 0.974 Theta(max)= 28.696

R(reflections)= 0.0449(8335) wR2(reflections)= 0.0943(9273)

S = 1.237 Npar= 447

The following ALERTS were generated. Each ALERT has the format
test-name_ALERT_alert-type_alert-level.
Click on the hyperlinks for more details of the test.

Alert level B

```
PLAT220_ALERT_2_B NonSolvent   Resd 1  C   Ueq(max)/Ueq(min) Range        6.3 Ratio
```

Alert level C

```
PLAT213_ALERT_2_C Atom C10             has ADP max/min Ratio .....        3.2 prolat
PLAT213_ALERT_2_C Atom C33             has ADP max/min Ratio .....        3.1 prolat
PLAT213_ALERT_2_C Atom C34             has ADP max/min Ratio .....        3.6 prolat
PLAT222_ALERT_3_C NonSolvent Resd 1  H   Uiso(max)/Uiso(min) Range        6.7 Ratio
PLAT241_ALERT_2_C High   'MainMol' Ueq as Compared to Neighbors of        C10 Check
PLAT242_ALERT_2_C Low    'MainMol' Ueq as Compared to Neighbors of        C31 Check
PLAT601_ALERT_2_C Unit Cell Contains Solvent Accessible VOIDS   <=         67 Ang**3
PLAT906_ALERT_3_C Large K Value in the Analysis of Variance ......      6.100 Check
PLAT911_ALERT_3_C Missing FCF Refl Between Thmin & STh/L=    0.600          8 Report
               10  1  0,   8  7  0,   2  0  2,   6  3  2,   8  3  2,   3  0  3,
                3  2  3,   6  5  6,
PLAT977_ALERT_2_C Check Negative Difference Density on H10       .      -0.34 eA-3
PLAT977_ALERT_2_C Check Negative Difference Density on H38       .      -0.33 eA-3
```

Alert level G

```
PLAT003_ALERT_2_G Number of Uiso or U(i,j)  Restrained non-H-Atoms         11 Report
PLAT066_ALERT_1_G Predicted and Reported Tmin&Tmax Range Identical          ? Check
PLAT068_ALERT_1_G Reported F000 Differs from Calcd (or Missing)...     Please Check
PLAT083_ALERT_2_G SHELXL Second Parameter in WGHT  Unusually Large      18.86 Why ?
PLAT177_ALERT_4_G The CIF-Embedded .res File Contains DELU Records          2 Report
PLAT178_ALERT_4_G The CIF-Embedded .res File Contains SIMU Records          1 Report
PLAT186_ALERT_4_G The CIF-Embedded .res File Contains ISOR Records          1 Report
PLAT188_ALERT_3_G A Non-default SIMU Restraint Value has been used     0.0100 Report
PLAT300_ALERT_4_G Atom Site Occupancy of S1         Constrained at        0.3 Check
PLAT300_ALERT_4_G Atom Site Occupancy of S2         Constrained at        0.3 Check
PLAT300_ALERT_4_G Atom Site Occupancy of S3         Constrained at        0.3 Check
PLAT300_ALERT_4_G Atom Site Occupancy of S4         Constrained at        0.3 Check
PLAT300_ALERT_4_G Atom Site Occupancy of S5         Constrained at        0.3 Check
PLAT300_ALERT_4_G Atom Site Occupancy of S6         Constrained at        0.3 Check
PLAT300_ALERT_4_G Atom Site Occupancy of S7         Constrained at        0.2 Check
PLAT300_ALERT_4_G Atom Site Occupancy of S8         Constrained at        0.2 Check
PLAT300_ALERT_4_G Atom Site Occupancy of S9         Constrained at        0.2 Check
PLAT300_ALERT_4_G Atom Site Occupancy of S10        Constrained at        0.2 Check
PLAT301_ALERT_3_G Main Residue  Disorder .............(Resd    1)         6% Note
PLAT304_ALERT_4_G Non-Integer Number of Atoms in ..... (Resd    1)     182.20 Check
PLAT343_ALERT_2_G Unusual sp?      Angle Range in Main Residue for         C1 Check
PLAT789_ALERT_4_G Atoms with Negative _atom_site_disorder_group  #         10 Check
PLAT794_ALERT_5_G Tentative Bond Valency for Fe1       (II)      .       2.11 Info
PLAT822_ALERT_4_G CIF-embedded .res Contains Negative PART Numbers          2 Check
PLAT860_ALERT_3_G Number of Least-Squares Restraints ............         156 Note
PLAT883_ALERT_1_G Absent Datum for _atom_sites_solution_primary ..     Please Do !
PLAT899_ALERT_4_G SHELXL2018 is Outdated   and Succeeded by SHELXL     2019/3 Note
PLAT910_ALERT_3_G Missing FCF Reflection(s) Below Theta(Min)[Deg]=       1.69 Note
                0  2  0,
PLAT912_ALERT_4_G Missing # of FCF Reflections Above STh/L=  0.600        237 Note
PLAT913_ALERT_3_G Missing # of Very Strong Reflections in FCF ....          1 Note
                0  2  0,
PLAT933_ALERT_2_G Number of HKL-OMIT Records in Embedded .res File          8 Note
                3  0  3,   3  2  3,   8  3  2,   8  7  0,  10  1  0,   6  3  2,
                6  5  6,   2  0  2,
PLAT965_ALERT_2_G The SHELXL WEIGHT Optimisation has not Converged     Please Check
PLAT969_ALERT_5_G The 'Henn et al.' R-Factor-gap value ..........       6.413 Note
              Predicted wR2: Based on SigI**2  1.47 or SHELX Weight  7.62
PLAT978_ALERT_2_G Number C-C Bonds with Positive Residual Density.          7 Info
```

```
   0 ALERT level A = Most likely a serious problem - resolve or explain
   1 ALERT level B = A potentially serious problem, consider carefully
  11 ALERT level C = Check. Ensure it is not caused by an omission or oversight
  34 ALERT level G = General information/check it is not something unexpected

   3 ALERT type 1 CIF construction/syntax error, inconsistent or missing data
  15 ALERT type 2 Indicator that the structure model may be wrong or deficient
   8 ALERT type 3 Indicator that the structure quality may be low
  18 ALERT type 4 Improvement, methodology, query or suggestion
   2 ALERT type 5 Informative message, check
```

Datablock: compound6

Bond precision: C-C = 0.0069 A Wavelength=0.71073

Cell:	a=25.7657(3)	b=11.3819(1)	c=25.9495(3)
	alpha=90	beta=111.185(1)	gamma=90
Temperature:	103 K		

	Calculated	Reported
Volume	7095.72(14)	7095.72(14)
Space group	P 21/n	P 21/n
Hall group	-P 2yn	-P 2yn
Moiety formula	C152 H196 Fe4 Se8.40 Sn2	0.9(C76 H98 Fe2 Se4 Sn),0.1(C76 H98 Fe2 Se6 Sn)
Sum formula	C152 H196 Fe4 Se8.40 Sn2	C76 H98 Fe2 Se4.20 Sn
Mr	3147.18	1573.56
Dx,g cm-3	1.473	1.473
Z	2	4
Mu (mm-1)	2.948	2.948
F000	3195.2	3195.0
F000'	3194.65	
h,k,lmax	31,14,32	31,14,32
Nref	13936	13930
Tmin,Tmax	0.838,0.915	0.719,0.917
Tmin'	0.702	

Correction method= # Reported T Limits: Tmin=0.719 Tmax=0.917
AbsCorr = MULTI-SCAN

Data completeness= 1.000 Theta(max)= 26.000

R(reflections)= 0.0480(12763) wR2(reflections)= 0.1132(13930)

S = 1.139 Npar= 913

The following ALERTS were generated. Each ALERT has the format
test-name_ALERT_alert-type_alert-level.
Click on the hyperlinks for more details of the test.

Alert level B

PLAT971_ALERT_2_B Check Calcd Resid. Dens. 0.95Ang From Fe1 2.62 eA-3

Alert level C

PLAT041_ALERT_1_C Calc. and Reported SumFormula Strings Differ Please Check
Calc: C152 H196 Fe4 Se8.40 Sn2
Rep.: C76 H98 Fe2 Se4.20 Sn
PLAT042_ALERT_1_C Calc. and Reported MoietyFormula Strings Differ Please Check

```
              Calc: C152 H196 Fe4 Se8.40 Sn2
              Rep.: 0.9(C76 H98 Fe2 Se4 Sn),0.1(C76 H98 Fe2 Se6 Sn
                    )
PLAT077_ALERT_4_C Unit Cell Contains Non-integer Number of Atoms .     Please Check
PLAT094_ALERT_2_C Ratio of Maximum / Minimum Residual Density ....       2.03 Report
PLAT213_ALERT_2_C Atom Se13            has ADP max/min Ratio .....        3.1 prolat
PLAT220_ALERT_2_C NonSolvent   Resd 1  C   Ueq(max)/Ueq(min) Range        3.6 Ratio
PLAT220_ALERT_2_C NonSolvent   Resd 1 Se   Ueq(max)/Ueq(min) Range        4.7 Ratio
PLAT222_ALERT_3_C NonSolvent Resd 1  H   Uiso(max)/Uiso(min) Range        4.1 Ratio
PLAT242_ALERT_2_C Low    'MainMol' Ueq as Compared to Neighbors of        C73 Check
PLAT303_ALERT_2_C Full Occupancy Atom H14       with # Connections       1.10 Check
PLAT906_ALERT_3_C Large K Value in the Analysis of Variance ......      4.224 Check
```

Alert level G

```
PLAT003_ALERT_2_G Number of Uiso or U(i,j)  Restrained non-H-Atoms          9 Report
PLAT045_ALERT_1_G Calculated and Reported Z Differ by a Factor ...      0.500 Check
PLAT068_ALERT_1_G Reported F000 Differs from Calcd (or Missing)...     Please Check
PLAT083_ALERT_2_G SHELXL Second Parameter in WGHT  Unusually Large      27.46 Why ?
PLAT142_ALERT_4_G s.u. on b - Axis Small or Missing .............     0.00010 Ang.
PLAT178_ALERT_4_G The CIF-Embedded .res File Contains SIMU Records          2 Report
PLAT188_ALERT_3_G A Non-default SIMU Restraint Value has been used     0.0100 Report
PLAT188_ALERT_3_G A Non-default SIMU Restraint Value has been used     0.0030 Report
PLAT230_ALERT_2_G Hirshfeld Test Diff for    Se1      --Se2      .       10.4 s.u.
PLAT230_ALERT_2_G Hirshfeld Test Diff for    Se3      --Se4      .        8.1 s.u.
PLAT230_ALERT_2_G Hirshfeld Test Diff for    C73      --C75      .        7.3 s.u.
PLAT230_ALERT_2_G Hirshfeld Test Diff for    C73      --C76      .        7.7 s.u.
PLAT230_ALERT_2_G Hirshfeld Test Diff for    C73      --C80      .       11.3 s.u.
PLAT230_ALERT_2_G Hirshfeld Test Diff for    C73      --C81      .        7.2 s.u.
PLAT230_ALERT_2_G Hirshfeld Test Diff for    C73      --C82      .        5.7 s.u.
PLAT232_ALERT_2_G Hirshfeld Test Diff (M-X)  Sn1      --Se1      .        9.5 s.u.
PLAT232_ALERT_2_G Hirshfeld Test Diff (M-X)  Sn2      --C1       .       12.0 s.u.
PLAT232_ALERT_2_G Hirshfeld Test Diff (M-X)  Fe1      --C4       .        6.0 s.u.
PLAT300_ALERT_4_G Atom Site Occupancy of Sn1        Constrained at        0.7 Check
PLAT300_ALERT_4_G Atom Site Occupancy of Sn2        Constrained at        0.2 Check
PLAT300_ALERT_4_G Atom Site Occupancy of Sn3        Constrained at        0.1 Check
PLAT300_ALERT_4_G Atom Site Occupancy of Se1        Constrained at        0.7 Check
PLAT300_ALERT_4_G Atom Site Occupancy of Se2        Constrained at        0.7 Check
PLAT300_ALERT_4_G Atom Site Occupancy of Se3        Constrained at        0.7 Check
PLAT300_ALERT_4_G Atom Site Occupancy of Se4        Constrained at        0.7 Check
PLAT300_ALERT_4_G Atom Site Occupancy of Se5        Constrained at        0.2 Check
PLAT300_ALERT_4_G Atom Site Occupancy of Se6        Constrained at        0.2 Check
PLAT300_ALERT_4_G Atom Site Occupancy of Se7        Constrained at        0.2 Check
PLAT300_ALERT_4_G Atom Site Occupancy of Se8        Constrained at        0.2 Check
PLAT300_ALERT_4_G Atom Site Occupancy of Se9        Constrained at        0.1 Check
PLAT300_ALERT_4_G Atom Site Occupancy of Se10       Constrained at        0.1 Check
PLAT300_ALERT_4_G Atom Site Occupancy of Se11       Constrained at        0.1 Check
PLAT300_ALERT_4_G Atom Site Occupancy of Se12       Constrained at        0.1 Check
PLAT300_ALERT_4_G Atom Site Occupancy of Se13       Constrained at        0.1 Check
PLAT300_ALERT_4_G Atom Site Occupancy of Se14       Constrained at        0.1 Check
PLAT301_ALERT_3_G Main Residue  Disorder .............(Resd    1)         13% Note
PLAT304_ALERT_4_G Non-Integer Number of Atoms in ..... (Resd    1)     362.40 Check
PLAT412_ALERT_2_G Short Intra XH3 .. XHn     H58      ..H105     .       2.03 Ang.
                                                      x,y,z  =      1_555 Check
PLAT412_ALERT_2_G Short Intra XH3 .. XHn     H79      ..H114     .       2.00 Ang.
                                                      x,y,z  =      1_555 Check
PLAT412_ALERT_2_G Short Intra XH3 .. XHn     H80      ..H90      .       2.06 Ang.
```

```
                                                     x,y,z  =      1_555 Check
PLAT412_ALERT_2_G Short Intra XH3 .. XHn     H80      ..H113     .       2.12 Ang.
                                                     x,y,z  =      1_555 Check
PLAT779_ALERT_4_G Suspect or Irrelevant (Bond) Angle(s) in CIF ...      44.70 Deg.
              SE10 -C19  -H14     3_666   1_555   1_555 ......     #  154 Check
PLAT794_ALERT_5_G Tentative Bond Valency for Fe1       (II)      .       2.09 Info
PLAT794_ALERT_5_G Tentative Bond Valency for Fe2       (II)      .       2.15 Info
PLAT802_ALERT_4_G CIF Input Record(s) with more than 80 Characters          1 Info
PLAT860_ALERT_3_G Number of Least-Squares Restraints ............          84 Note
PLAT883_ALERT_1_G Absent Datum for _atom_sites_solution_primary ..     Please Do !
PLAT899_ALERT_4_G SHELXL2018 is Outdated   and Succeeded by SHELXL     2019/3 Note
PLAT910_ALERT_3_G Missing FCF Reflection(s) Below Theta(Min)[Deg]=       1.91 Note
                2  0  0,  -1  0  1,   1  0  1,   0  0  2,
PLAT913_ALERT_3_G Missing # of Very Strong Reflections in FCF ....          1 Note
                2  0  0,
PLAT960_ALERT_3_G Number of Intensities with I < - 2*Sigma(I) ....          2 Check
PLAT967_ALERT_5_G Note: Two-Theta Cutoff Value in Embedded .res ..       52.0 Degree
PLAT969_ALERT_5_G The 'Henn et al.' R-Factor-gap value ..........      9.076 Note
              Predicted wR2: Based on SigI**2  1.25 or SHELX Weight  9.94
PLAT978_ALERT_2_G Number C-C Bonds with Positive Residual Density.          2 Info
```

---

0 **ALERT level A** = Most likely a serious problem - resolve or explain
1 **ALERT level B** = A potentially serious problem, consider carefully
11 **ALERT level C** = Check. Ensure it is not caused by an omission or oversight
54 **ALERT level G** = General information/check it is not something unexpected

5 ALERT type 1 CIF construction/syntax error, inconsistent or missing data
24 ALERT type 2 Indicator that the structure model may be wrong or deficient
9 ALERT type 3 Indicator that the structure quality may be low
24 ALERT type 4 Improvement, methodology, query or suggestion
4 ALERT type 5 Informative message, check

Datablock: compound7

Bond precision: C-C = 0.0037 A Wavelength=0.71073

Cell:	a=12.6041(1)	b=13.7615(1)	c=23.5222(2)
	alpha=97.278(1)	beta=103.250(1)	gamma=100.371(1)
Temperature:	103 K		

	Calculated	Reported
Volume	3845.90(6)	3845.90(6)
Space group	P -1	P -1
Hall group	-P 1	-P 1
Moiety formula	C81.57 H106.72 Fe2 S Sn, C6 H6, 0.426(C H3)	C82 H108 Fe2 S Sn, C6 H6
Sum formula	C88 H114 Fe2 S Sn	C88 H114 Fe2 S Sn
Mr	1434.27	1434.24
Dx,g cm-3	1.239	1.239
Z	2	2
Mu (mm-1)	0.765	0.765
F000	1520.0	1520.0
F000'	1520.72	
h,k,lmax	17,18,32	17,18,32
Nref	20726	19331
Tmin,Tmax	0.929,0.962	0.894,0.963
Tmin'	0.892	

Correction method= # Reported T Limits: Tmin=0.894 Tmax=0.963
AbsCorr = MULTI-SCAN

Data completeness= 0.933 Theta(max)= 29.141

R(reflections)= 0.0363(18272) wR2(reflections)= 0.0943(19331)

S = 1.046 Npar= 905

The following ALERTS were generated. Each ALERT has the format
test-name_ALERT_alert-type_alert-level.
Click on the hyperlinks for more details of the test.

Alert level B

```
PLAT230_ALERT_2_B Hirshfeld Test Diff for    S1      --C4       .        7.2 s.u.
PLAT230_ALERT_2_B Hirshfeld Test Diff for    C1      --C2       .        7.2 s.u.
```

Alert level C

```
PLAT042_ALERT_1_C Calc. and Reported MoietyFormula Strings  Differ     Please Check
              Calc: C81.57 H106.72 Fe2 S Sn, C6 H6, 0.426(C H3)
              Rep.: C82 H108 Fe2 S Sn, C6 H6
PLAT094_ALERT_2_C Ratio of Maximum / Minimum Residual Density ....       3.33 Report
PLAT220_ALERT_2_C NonSolvent   Resd 1  C   Ueq(max)/Ueq(min) Range        5.1 Ratio
PLAT222_ALERT_3_C NonSolvent Resd 1  H   Uiso(max)/Uiso(min) Range        5.4 Ratio
PLAT230_ALERT_2_C Hirshfeld Test Diff for    C2      --C5       .        6.8 s.u.
PLAT230_ALERT_2_C Hirshfeld Test Diff for    C61     --C63      .        5.3 s.u.
PLAT241_ALERT_2_C High   'MainMol' Ueq as Compared to Neighbors of       C82 Check
PLAT242_ALERT_2_C Low    'MainMol' Ueq as Compared to Neighbors of       C23 Check
```

```
PLAT242_ALERT_2_C Low    'MainMol' Ueq as Compared to Neighbors of        C61 Check
PLAT331_ALERT_2_C Small Aver Phenyl C-C Dist C89      --C94      .       1.36 Ang.
PLAT601_ALERT_2_C Unit Cell Contains Solvent Accessible VOIDS    <=         64 Ang**3
PLAT910_ALERT_3_C Missing FCF Reflection(s) Below Theta(Min)[Deg]=       1.91 Note
                1  0  0,   0  1  0,   0 -1  1,  -1  0  1,   0  0  1,   0  0  2,
PLAT911_ALERT_3_C Missing FCF Refl Between Thmin & STh/L=    0.600         14 Report
               -2-15 11,  -2-14 13,  -1-15 14,  -2-13 14,  -3-14 15,  -2-14 15,
               -2-13 15,  -3-14 16,  -2-14 16,  -3-13 16,  -2-13 16,  -2-14 17,
               -3-13 17,  -3-13 18,
PLAT918_ALERT_3_C Reflection(s) with I(obs) much Smaller I(calc) .          2 Check
PLAT971_ALERT_2_C Check Calcd Resid. Dens.  0.93Ang From Fe2            2.29 eA-3
PLAT971_ALERT_2_C Check Calcd Resid. Dens.  1.58Ang From C3             1.81 eA-3
PLAT971_ALERT_2_C Check Calcd Resid. Dens.  0.78Ang From Sn1            1.59 eA-3
PLAT977_ALERT_2_C Check Negative Difference Density on H2        .      -0.32 eA-3
```

Alert level G

```
PLAT066_ALERT_1_G Predicted and Reported Tmin&Tmax Range Identical          ? Check
PLAT083_ALERT_2_G SHELXL Second Parameter in WGHT  Unusually Large       5.48 Why ?
PLAT154_ALERT_1_G The s.u.'s on the Cell Angles are Equal ..(Note)      0.001 Degree
PLAT230_ALERT_2_G Hirshfeld Test Diff for    C65      --C68      .        9.3 s.u.
PLAT230_ALERT_2_G Hirshfeld Test Diff for    C82      --C83      .       22.2 s.u.
PLAT230_ALERT_2_G Hirshfeld Test Diff for    C82      --C85      .       15.0 s.u.
PLAT230_ALERT_2_G Hirshfeld Test Diff for    C82      --C86      .       10.2 s.u.
PLAT230_ALERT_2_G Hirshfeld Test Diff for    C82      --C88      .       10.8 s.u.
PLAT232_ALERT_2_G Hirshfeld Test Diff (M-X)  Sn1      --S1       .        8.0 s.u.
PLAT301_ALERT_3_G Main Residue  Disorder .............(Resd    1)          7% Note
PLAT302_ALERT_4_G Anion/Solvent/Minor-Residue Disorder (Resd    3)        100% Note
PLAT304_ALERT_4_G Non-Integer Number of Atoms in ..... (Resd    1)     192.30 Check
PLAT304_ALERT_4_G Non-Integer Number of Atoms in ..... (Resd    3)       1.70 Check
PLAT343_ALERT_2_G Unusual sp?      Angle Range in Main Residue for         C7 Check
PLAT343_ALERT_2_G Unusual sp?      Angle Range in Main Residue for        C45 Check
PLAT367_ALERT_2_G Long?  C(sp?)-C(sp?) Bond  C76      - C82      .       1.52 Ang.
PLAT412_ALERT_2_G Short Intra XH3 .. XHn     H68      ..H82      .       2.06 Ang.
                                                      x,y,z  =     1_555 Check
PLAT412_ALERT_2_G Short Intra XH3 .. XHn     H98      ..H112     .       2.08 Ang.
                                                      x,y,z  =     1_555 Check
PLAT412_ALERT_2_G Short Intra XH3 .. XHn     H98      ..H115     .       2.04 Ang.
                                                      x,y,z  =     1_555 Check
PLAT412_ALERT_2_G Short Intra XH3 .. XHn     H99      ..H118     .       1.97 Ang.
                                                      x,y,z  =     1_555 Check
PLAT432_ALERT_2_G Short Inter X...Y Contact  C76      ..C87      .       2.61 Ang.
                                                      x,y,z  =     1_555 Check
PLAT432_ALERT_2_G Short Inter X...Y Contact  C77      ..C87      .       3.05 Ang.
                                                      x,y,z  =     1_555 Check
PLAT432_ALERT_2_G Short Inter X...Y Contact  C82      ..C87      .       1.77 Ang.
                                                      x,y,z  =     1_555 Check
PLAT773_ALERT_2_G Check long C-C Bond in CIF: C65      --C69             1.73 Ang.
PLAT773_ALERT_2_G Check long C-C Bond in CIF: C82      --C86             1.74 Ang.
PLAT773_ALERT_2_G Check long C-C Bond in CIF: C82      --C87             1.77 Ang.
PLAT794_ALERT_5_G Tentative Bond Valency for Fe1       (II)      .       2.09 Info
PLAT794_ALERT_5_G Tentative Bond Valency for Fe2       (II)      .       2.10 Info
PLAT883_ALERT_1_G Absent Datum for _atom_sites_solution_primary ..     Please Do !
PLAT899_ALERT_4_G SHELXL2018 is Outdated   and Succeeded by SHELXL     2019/3 Note
PLAT912_ALERT_4_G Missing # of FCF Reflections Above STh/L=  0.600       1373 Note
PLAT941_ALERT_3_G Average HKL Measurement Multiplicity ..........        3.4 Low
PLAT969_ALERT_5_G The 'Henn et al.' R-Factor-gap value ..........     11.378 Note
```

```
            Predicted wR2: Based on SigI**2  0.83 or SHELX Weight  9.02
PLAT978_ALERT_2_G Number C-C Bonds with Positive Residual Density.          4 Info
```

```
   0 ALERT level A = Most likely a serious problem - resolve or explain
   2 ALERT level B = A potentially serious problem, consider carefully
  18 ALERT level C = Check. Ensure it is not caused by an omission or oversight
  34 ALERT level G = General information/check it is not something unexpected

   4 ALERT type 1 CIF construction/syntax error, inconsistent or missing data
  36 ALERT type 2 Indicator that the structure model may be wrong or deficient
   6 ALERT type 3 Indicator that the structure quality may be low
   5 ALERT type 4 Improvement, methodology, query or suggestion
   3 ALERT type 5 Informative message, check
```

Datablock: compound8

```
Bond precision:    C-C = 0.0030 A          Wavelength=0.71073

Cell:              a=11.5071(1)     b=16.2370(2)        c=20.2036(2)
                   alpha=90         beta=104.665(1)     gamma=90
Temperature:       103 K

                  Calculated                         Reported
Volume            3651.88(7)                         3651.88(7)
Space group       P 2/c                              P 2/c
Hall group        -P 2yc                             -P 2yc
Moiety formula    C76 H100 Fe2 O S Sn, C6 H14 C76 H100 Fe2 O S Sn, C6 H14
Sum formula       C82 H114 Fe2 O S Sn                C82 H114 Fe2 O S Sn
Mr                1378.20                            1378.18
Dx,g cm-3         1.253                              1.253
Z                 2                                  2
Mu (mm-1)         0.803                              0.803
F000              1464.0                             1464.0
F000'             1464.72
h,k,lmax          14,20,25                           14,20,25
Nref              7981                               7974
Tmin,Tmax         0.926,0.961                        0.889,0.961
Tmin'             0.887

Correction method= # Reported T Limits: Tmin=0.889 Tmax=0.961
AbsCorr = MULTI-SCAN

Data completeness= 0.999            Theta(max)= 26.999
```

R(reflections)= 0.0315(7387)

wR2(reflections)= 0.0780(7974)

S = 1.068 Npar= 541

The following ALERTS were generated. Each ALERT has the format
test-name_ALERT_alert-type_alert-level.
Click on the hyperlinks for more details of the test.

Alert level B

PLAT250_ALERT_2_B Large U3/U1 Ratio for <U(i,j)> Tensor(Resd 2) 4.2 Note

---

Alert level C

PLAT213_ALERT_2_C Atom C41 has ADP max/min Ratio ..... 3.4 prolat
PLAT213_ALERT_2_C Atom C44 has ADP max/min Ratio ..... 3.6 prolat
PLAT220_ALERT_2_C NonSolvent Resd 1 C Ueq(max)/Ueq(min) Range 3.6 Ratio
PLAT222_ALERT_3_C NonSolvent Resd 1 H Uiso(max)/Uiso(min) Range 4.3 Ratio
PLAT234_ALERT_4_C Large Hirshfeld Difference C31 --C41 . 0.19 Ang.
PLAT242_ALERT_2_C Low 'MainMol' Ueq as Compared to Neighbors of C21 Check
PLAT250_ALERT_2_C Large U3/U1 Ratio for <U(i,j)> Tensor(Resd 3) 4.0 Note
PLAT260_ALERT_2_C Large Average Ueq of Residue Including C45 0.148 Check
PLAT260_ALERT_2_C Large Average Ueq of Residue Including C51 0.146 Check
PLAT420_ALERT_2_C D-H Bond Without Acceptor S1 --H1 . Please Check
PLAT906_ALERT_3_C Large K Value in the Analysis of Variance ...... 2.208 Check
PLAT911_ALERT_3_C Missing FCF Refl Between Thmin & STh/L= 0.600 4 Report
6 3 3, -2 0 6, -6 3 9, -3 0 18,

Alert level G

PLAT002_ALERT_2_G Number of Distance or Angle Restraints on AtSite 12 Note
PLAT003_ALERT_2_G Number of Uiso or U(i,j) Restrained non-H-Atoms 12 Report
PLAT007_ALERT_5_G Number of Unrefined Donor-H Atoms .... 2 Report
H1 H2
PLAT066_ALERT_1_G Predicted and Reported Tmin&Tmax Range Identical ? Check
PLAT143_ALERT_4_G s.u. on c - Axis Small or Missing .... 0.00020 Ang.
PLAT172_ALERT_4_G The CIF-Embedded .res File Contains DFIX Records 4 Report
PLAT178_ALERT_4_G The CIF-Embedded .res File Contains SIMU Records 1 Report
PLAT188_ALERT_3_G A Non-default SIMU Restraint Value has been used 0.0100 Report
PLAT230_ALERT_2_G Hirshfeld Test Diff for C31 --C33 . 5.5 s.u.
PLAT232_ALERT_2_G Hirshfeld Test Diff (M-X) Sn1 --S1 . 42.6 s.u.
PLAT232_ALERT_2_G Hirshfeld Test Diff (M-X) Sn1 --O1 . 10.6 s.u.
PLAT299_ALERT_4_G Atom Site Occupancy Constrained at .... 0.5 Check
S1 O1 H1 H2
PLAT300_ALERT_4_G Atom Site Occupancy of C45 Constrained at 0.25 Check
PLAT300_ALERT_4_G Atom Site Occupancy of C46 Constrained at 0.25 Check
PLAT300_ALERT_4_G Atom Site Occupancy of C47 Constrained at 0.25 Check
PLAT300_ALERT_4_G Atom Site Occupancy of C48 Constrained at 0.25 Check
PLAT300_ALERT_4_G Atom Site Occupancy of C49 Constrained at 0.25 Check
PLAT300_ALERT_4_G Atom Site Occupancy of C50 Constrained at 0.25 Check
PLAT300_ALERT_4_G Atom Site Occupancy of H70 Constrained at 0.25 Check
PLAT300_ALERT_4_G Atom Site Occupancy of H71 Constrained at 0.25 Check
PLAT300_ALERT_4_G Atom Site Occupancy of H72 Constrained at 0.25 Check
PLAT300_ALERT_4_G Atom Site Occupancy of H73 Constrained at 0.25 Check
PLAT300_ALERT_4_G Atom Site Occupancy of H74 Constrained at 0.25 Check

```
PLAT300_ALERT_4_G Atom Site Occupancy of H75       Constrained at       0.25 Check
PLAT300_ALERT_4_G Atom Site Occupancy of H76       Constrained at       0.25 Check
PLAT300_ALERT_4_G Atom Site Occupancy of H77       Constrained at       0.25 Check
PLAT300_ALERT_4_G Atom Site Occupancy of H78       Constrained at       0.25 Check
PLAT300_ALERT_4_G Atom Site Occupancy of H79       Constrained at       0.25 Check
PLAT300_ALERT_4_G Atom Site Occupancy of H80       Constrained at       0.25 Check
PLAT300_ALERT_4_G Atom Site Occupancy of H81       Constrained at       0.25 Check
PLAT300_ALERT_4_G Atom Site Occupancy of H82       Constrained at       0.25 Check
PLAT300_ALERT_4_G Atom Site Occupancy of H83       Constrained at       0.25 Check
PLAT300_ALERT_4_G Atom Site Occupancy of C51       Constrained at       0.25 Check
PLAT300_ALERT_4_G Atom Site Occupancy of C52       Constrained at       0.25 Check
PLAT300_ALERT_4_G Atom Site Occupancy of C53       Constrained at       0.25 Check
PLAT300_ALERT_4_G Atom Site Occupancy of C54       Constrained at       0.25 Check
PLAT300_ALERT_4_G Atom Site Occupancy of C55       Constrained at       0.25 Check
PLAT300_ALERT_4_G Atom Site Occupancy of C56       Constrained at       0.25 Check
PLAT300_ALERT_4_G Atom Site Occupancy of H84       Constrained at       0.25 Check
PLAT300_ALERT_4_G Atom Site Occupancy of H85       Constrained at       0.25 Check
PLAT300_ALERT_4_G Atom Site Occupancy of H86       Constrained at       0.25 Check
PLAT300_ALERT_4_G Atom Site Occupancy of H87       Constrained at       0.25 Check
PLAT300_ALERT_4_G Atom Site Occupancy of H88       Constrained at       0.25 Check
PLAT300_ALERT_4_G Atom Site Occupancy of H89       Constrained at       0.25 Check
PLAT300_ALERT_4_G Atom Site Occupancy of H90       Constrained at       0.25 Check
PLAT300_ALERT_4_G Atom Site Occupancy of H91       Constrained at       0.25 Check
PLAT300_ALERT_4_G Atom Site Occupancy of H92       Constrained at       0.25 Check
PLAT300_ALERT_4_G Atom Site Occupancy of H93       Constrained at       0.25 Check
PLAT300_ALERT_4_G Atom Site Occupancy of H94       Constrained at       0.25 Check
PLAT300_ALERT_4_G Atom Site Occupancy of H95       Constrained at       0.25 Check
PLAT300_ALERT_4_G Atom Site Occupancy of H96       Constrained at       0.25 Check
PLAT300_ALERT_4_G Atom Site Occupancy of H97       Constrained at       0.25 Check
PLAT301_ALERT_3_G Main Residue  Disorder .............(Resd    1)        17% Note
PLAT302_ALERT_4_G Anion/Solvent/Minor-Residue Disorder (Resd    2)       100% Note
PLAT302_ALERT_4_G Anion/Solvent/Minor-Residue Disorder (Resd    3)       100% Note
PLAT343_ALERT_2_G Unusual sp?      Angle Range in Main Residue for         C1 Check
PLAT412_ALERT_2_G Short Intra XH3 .. XHn     H11      ..H25      .       2.14 Ang.
                                                 x,y,z  =      1_555 Check
PLAT413_ALERT_2_G Short Inter XH3 .. XHn     H50      ..H53      .       2.12 Ang.
                                            2-x,-y,1-z  =      3_756 Check
PLAT789_ALERT_4_G Atoms with Negative _atom_site_disorder_group  #         44 Check
PLAT794_ALERT_5_G Tentative Bond Valency for Fe1       (II)      .       2.08 Info
PLAT811_ALERT_5_G No ADDSYM Analysis: Too Many Excluded Atoms ....          ! Info
PLAT822_ALERT_4_G CIF-embedded .res Contains Negative PART Numbers          3 Check
PLAT860_ALERT_3_G Number of Least-Squares Restraints ............         216 Note
PLAT883_ALERT_1_G Absent Datum for _atom_sites_solution_primary ..     Please Do !
PLAT899_ALERT_4_G SHELXL2018 is Outdated   and Succeeded by SHELXL     2019/3 Note
PLAT910_ALERT_3_G Missing FCF Reflection(s) Below Theta(Min)[Deg]=       2.08 Note
                1  0  0,   0  1  0,   0  1  1,
PLAT933_ALERT_2_G Number of HKL-OMIT Records in Embedded .res File          5 Note
                1  0  0,  -6  3  9,   6  3  3,  -3  0 18,  -2  0  6,
PLAT967_ALERT_5_G Note: Two-Theta Cutoff Value in Embedded .res ..       54.0 Degree
PLAT969_ALERT_5_G The 'Henn et al.' R-Factor-gap value ..........       6.305 Note
              Predicted wR2: Based on SigI**2  1.24 or SHELX Weight  7.30
PLAT978_ALERT_2_G Number C-C Bonds with Positive Residual Density.         11 Info
```

---

0 **ALERT level A** = Most likely a serious problem - resolve or explain
1 **ALERT level B** = A potentially serious problem, consider carefully
12 **ALERT level C** = Check. Ensure it is not caused by an omission or oversight

```
70 ALERT level G = General information/check it is not something unexpected

 2 ALERT type 1 CIF construction/syntax error, inconsistent or missing data
19 ALERT type 2 Indicator that the structure model may be wrong or deficient
 7 ALERT type 3 Indicator that the structure quality may be low
50 ALERT type 4 Improvement, methodology, query or suggestion
 5 ALERT type 5 Informative message, check
```

It is advisable to attempt to resolve as many as possible of the alerts in all categories. Often the minor alerts point to easily fixed oversights, errors and omissions in your CIF or refinement strategy, so attention to these fine details can be worthwhile. In order to resolve some of the more serious problems it may be necessary to carry out additional measurements or structure refinements. However, the purpose of your study may justify the reported deviations and the more serious of these should normally be commented upon in the discussion or experimental section of a paper or in the "special_details" fields of the CIF. checkCIF was carefully designed to identify outliers and unusual parameters, but every test has its limitations and alerts that are not important in a particular case may appear. Conversely, the absence of alerts does not guarantee there are no aspects of the results needing attention. It is up to the individual to critically assess their own results and, if necessary, seek expert advice.

Publication of your CIF in IUCr journals

A basic structural check has been run on your CIF. These basic checks will be run on all CIFs submitted for publication in IUCr journals (*Acta Crystallographica*, *Journal of Applied Crystallography*, *Journal of Synchrotron Radiation*); however, if you intend to submit to *Acta Crystallographica Section C* or *E* or *IUCrData*, you should make sure that full publication checks are run on the final version of your CIF prior to submission.

Publication of your CIF in other journals

Please refer to the *Notes for Authors* of the relevant journal for any special instructions relating to CIF submission.

PLATON version of 04/06/2025; check.def file version of 30/05/2025

Datablock compound2 - ellipsoid plot

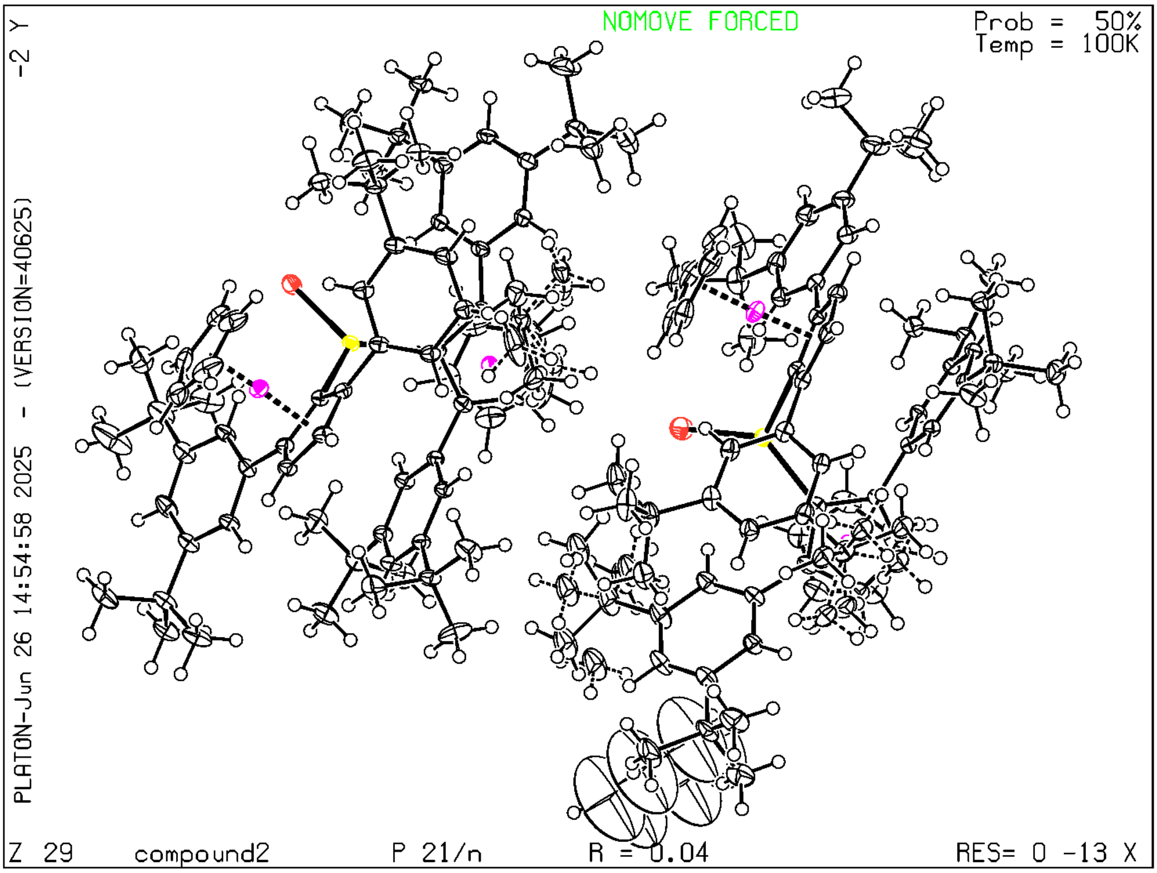

Datablock compound4a - ellipsoid plot

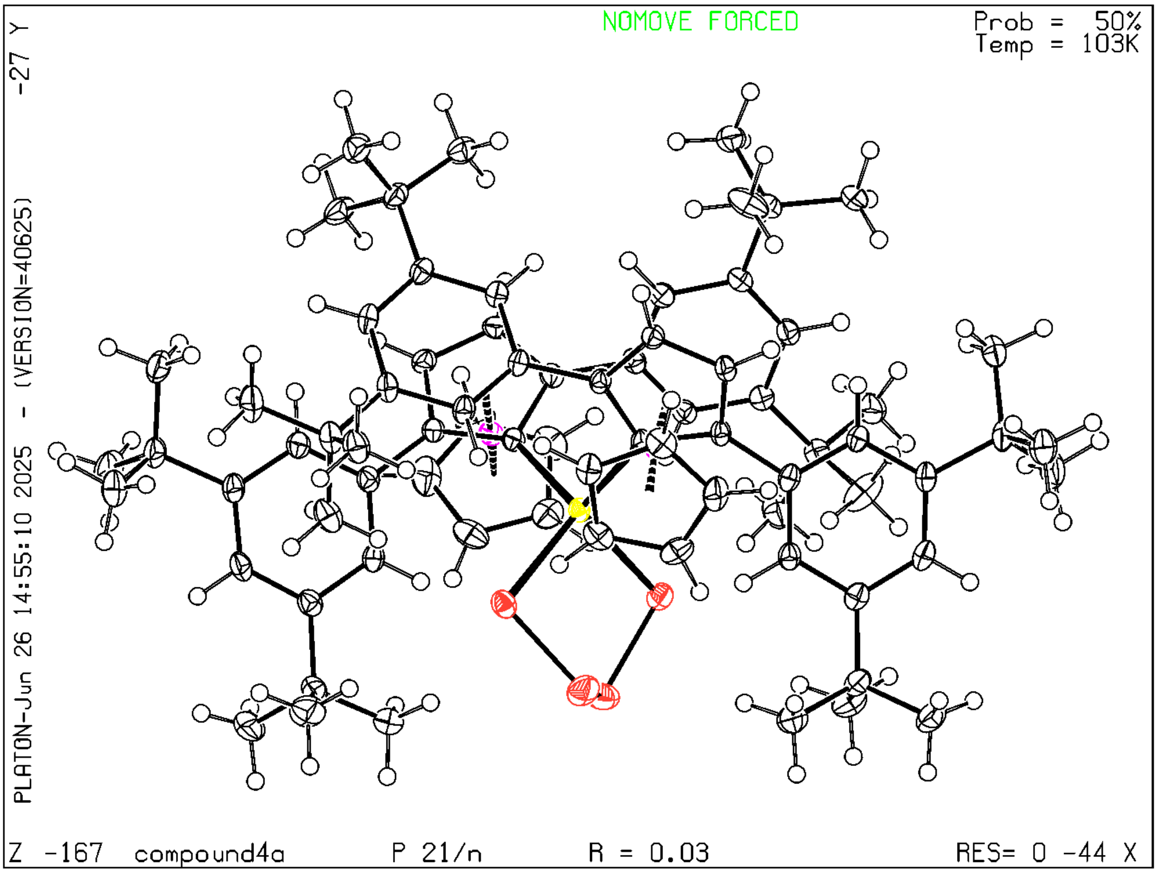

Datablock compound4b - ellipsoid plot

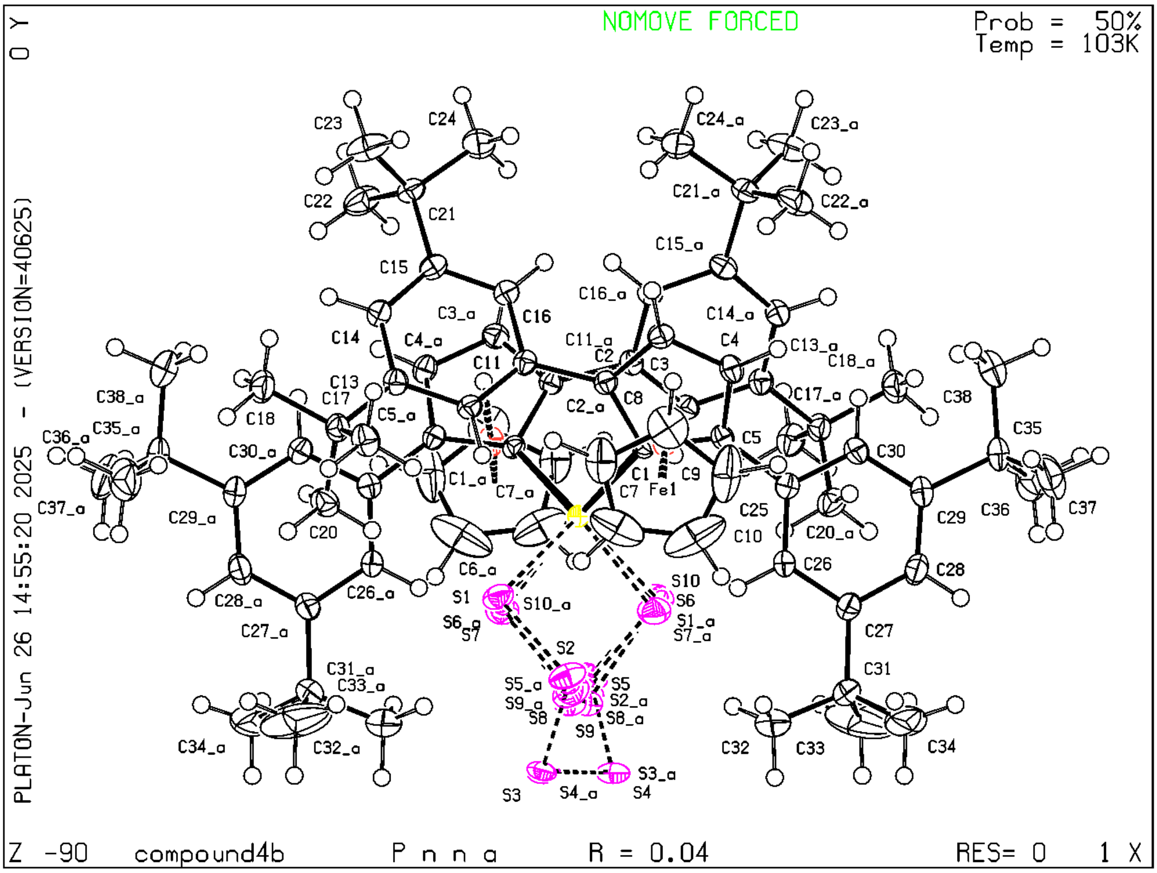

Datablock compound6 - ellipsoid plot

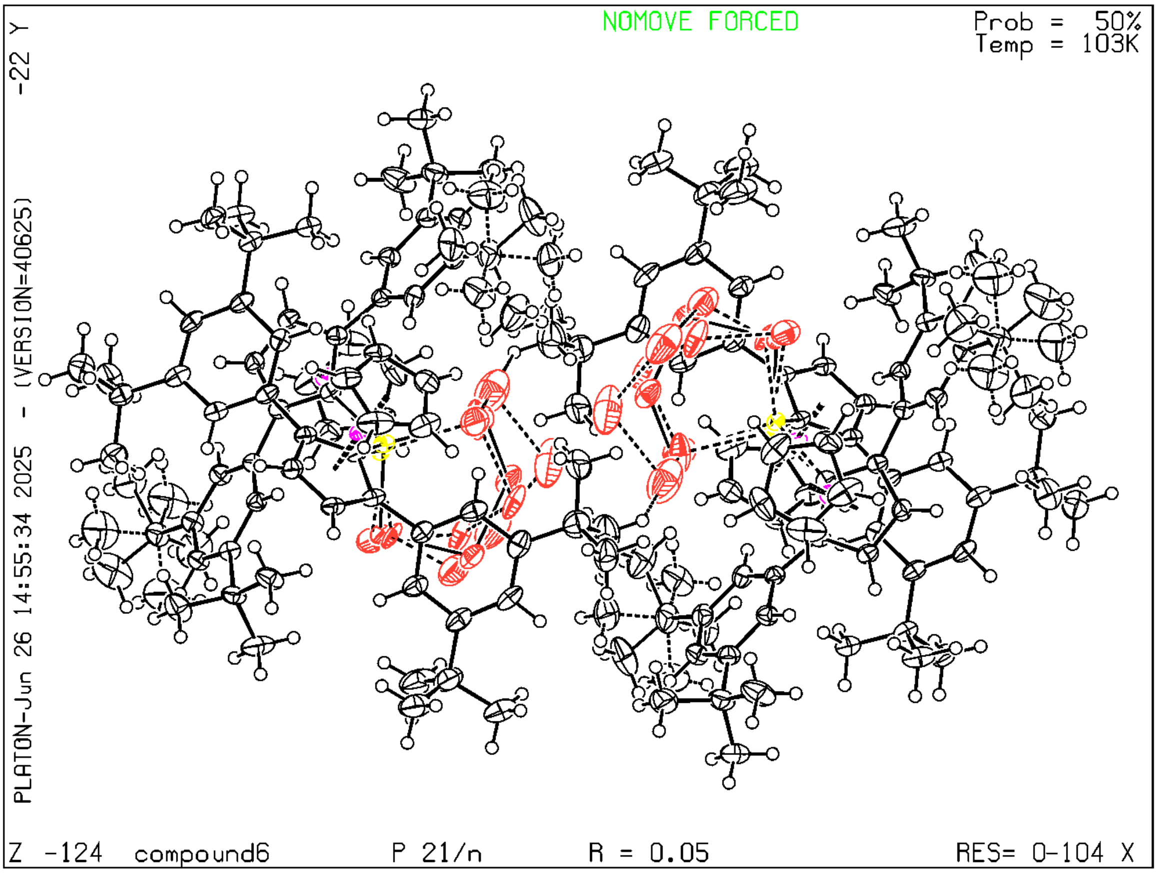

Datablock compound7 - ellipsoid plot

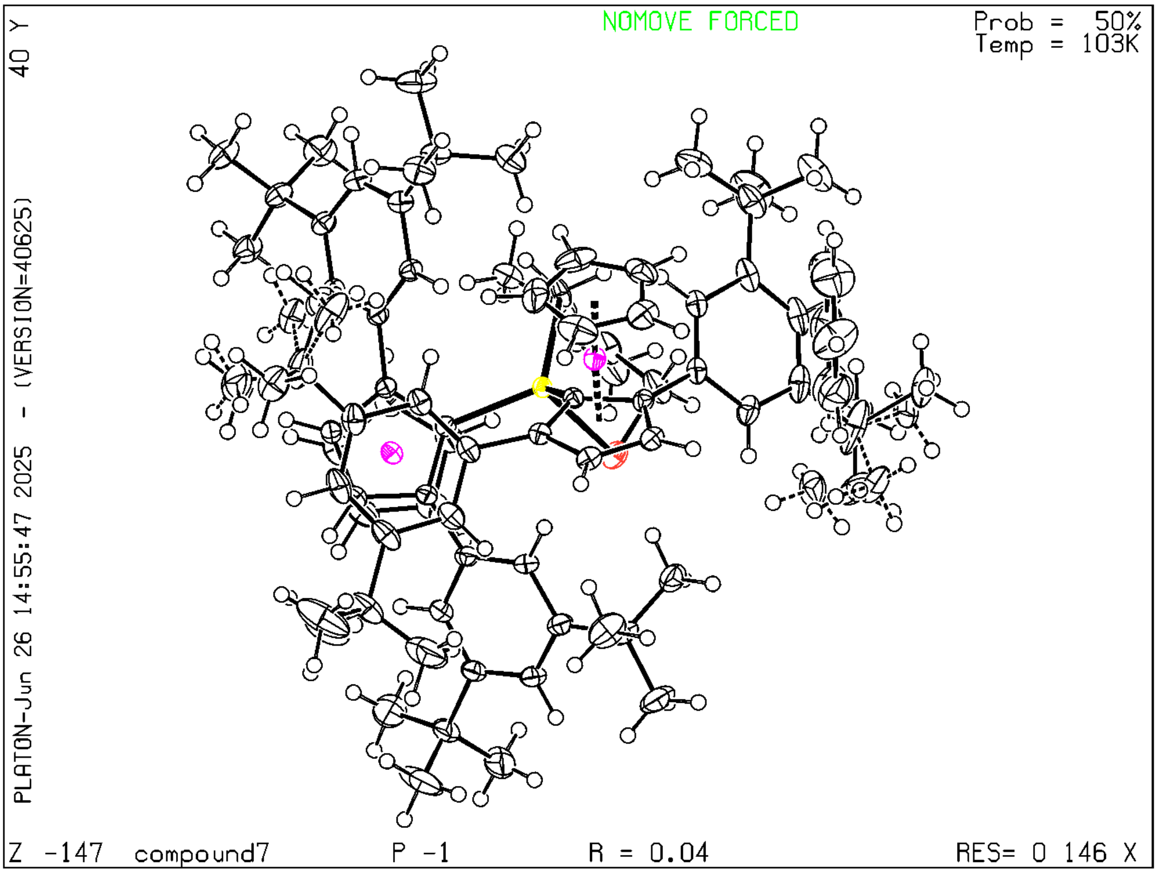

Datablock compound8 - ellipsoid plot

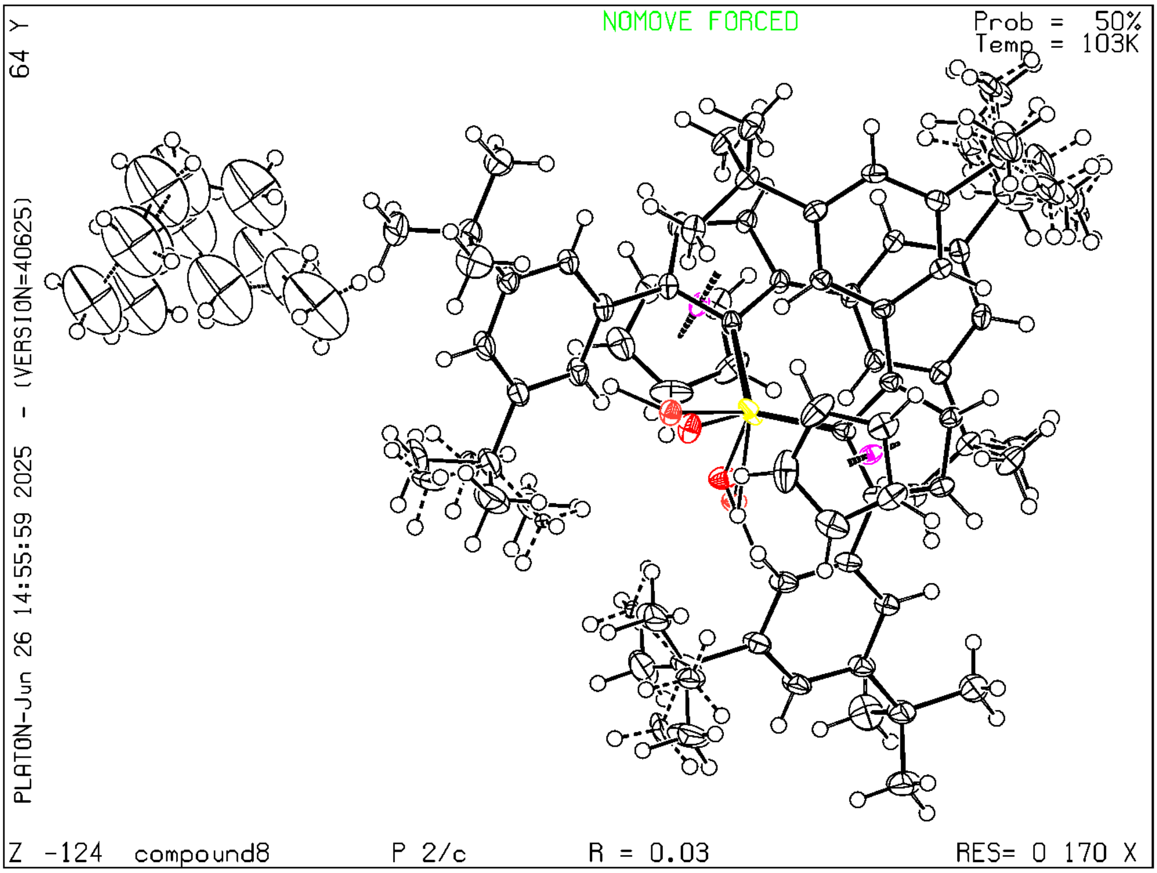

Supplement: Supplementary file 1 [file molecules-30-02826-s001.zip › molecules-3734450-supplementary/checkcifrev.pdf]
