# Supplementary material for: Ferrocenyl Substituted Stannanethione and Stannaneselone
Source: Molecules. 2025 Jun 30;30(13):2826. doi: 10.3390/molecules30132826 (PMC12250751; doi:10.3390/molecules30132826)
Supplement: Supplementary file 1 [file molecules-30-02826-s001.zip › molecules-3734450-supplementary/SnSe_SI_rev1.pdf]

Supporting Information

Ferrocenyl Substituted Stannanethione and Stannaneselone

Keisuke Iijima,[1] Koh Sugamata,[1,2] Takahiro Sasamori[1,2]

E-mail: sasamori@chem.tsukuba.ac.jp

Table of contents

NMR Spectrum 2

X-Ray Crystallographic Data 14

·References 14

NMR Spectrum

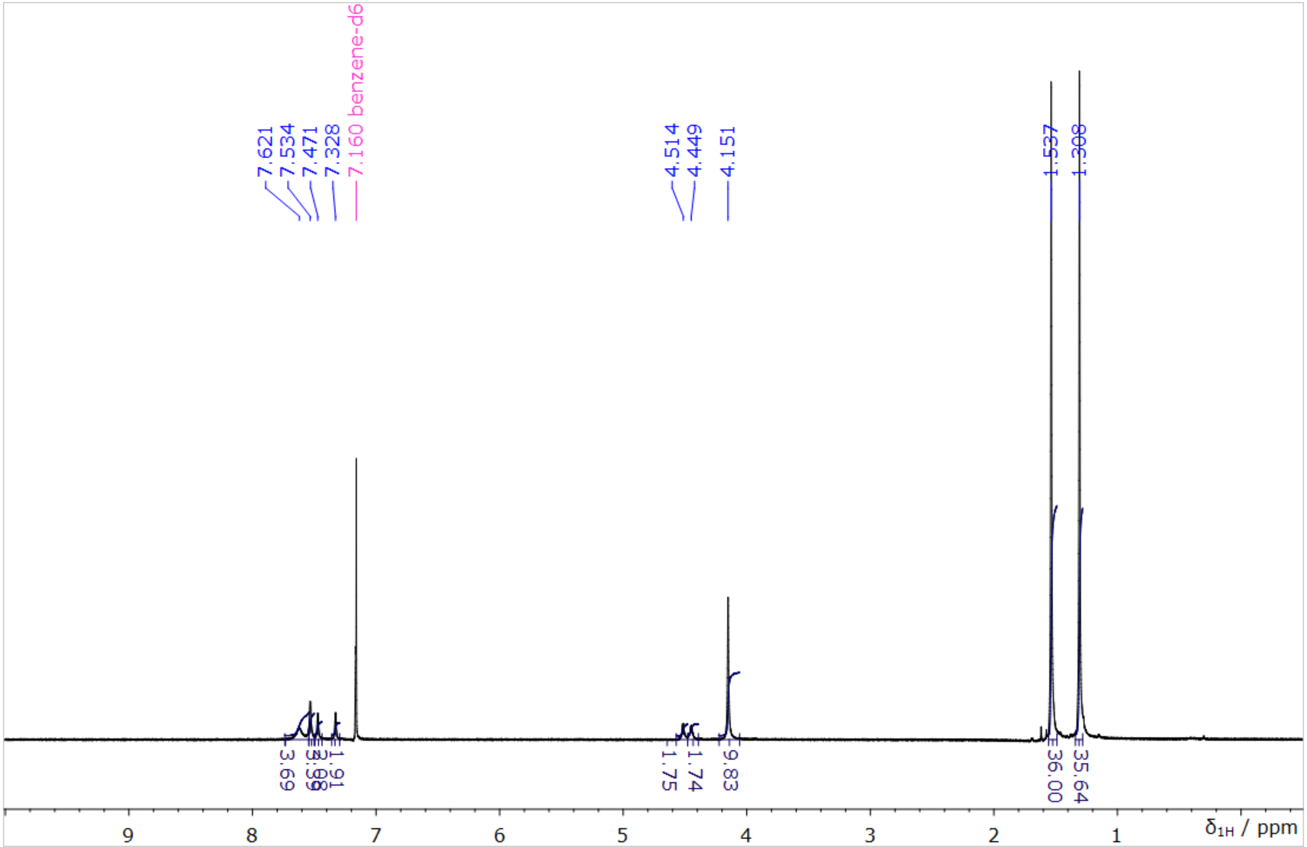

Figure S1. ^{1}H NMR spectrum of Bis(ferrocenyl)tetrathiastannolane **4a** in C_6D_6.

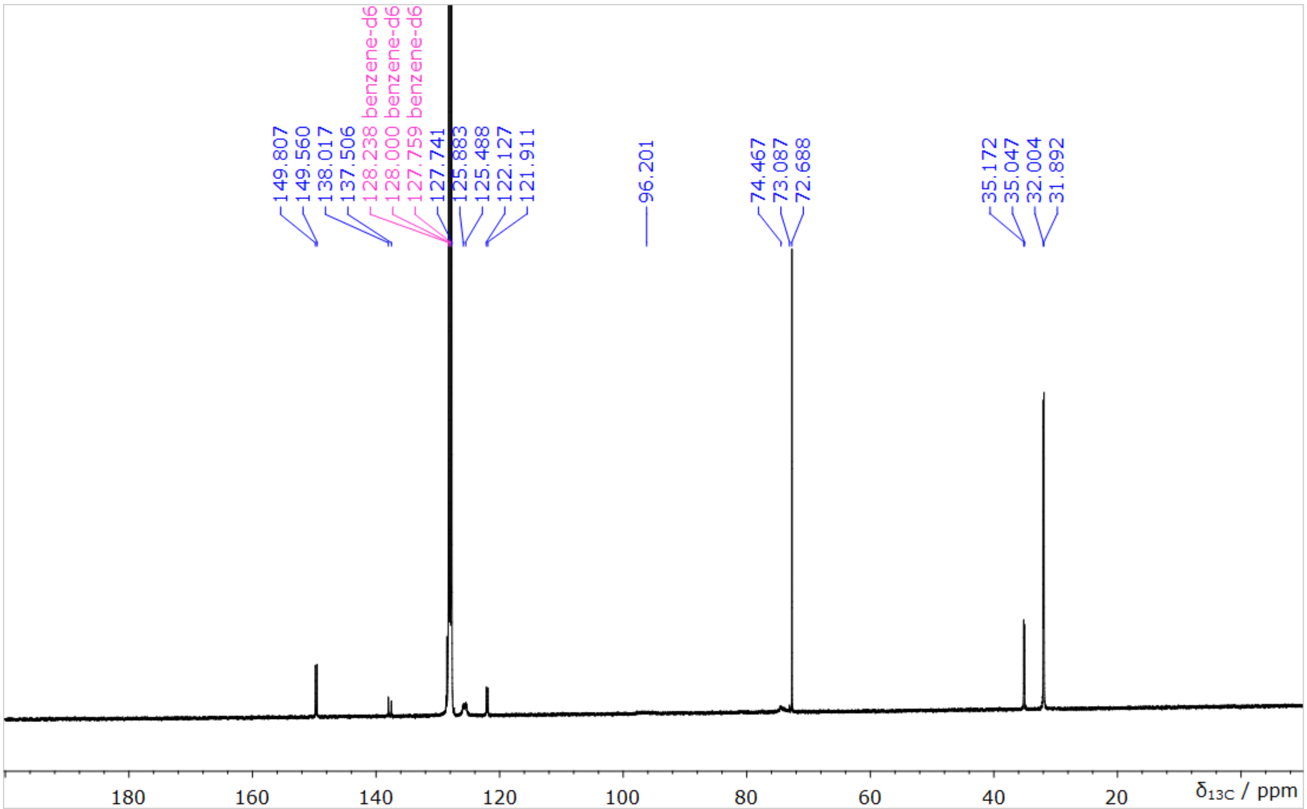

Figure S2. $^{13}C\{^1H\}$ NMR spectrum of Bis(ferrocenyl)tetrathiastannolane **4a** in C_6D_6.

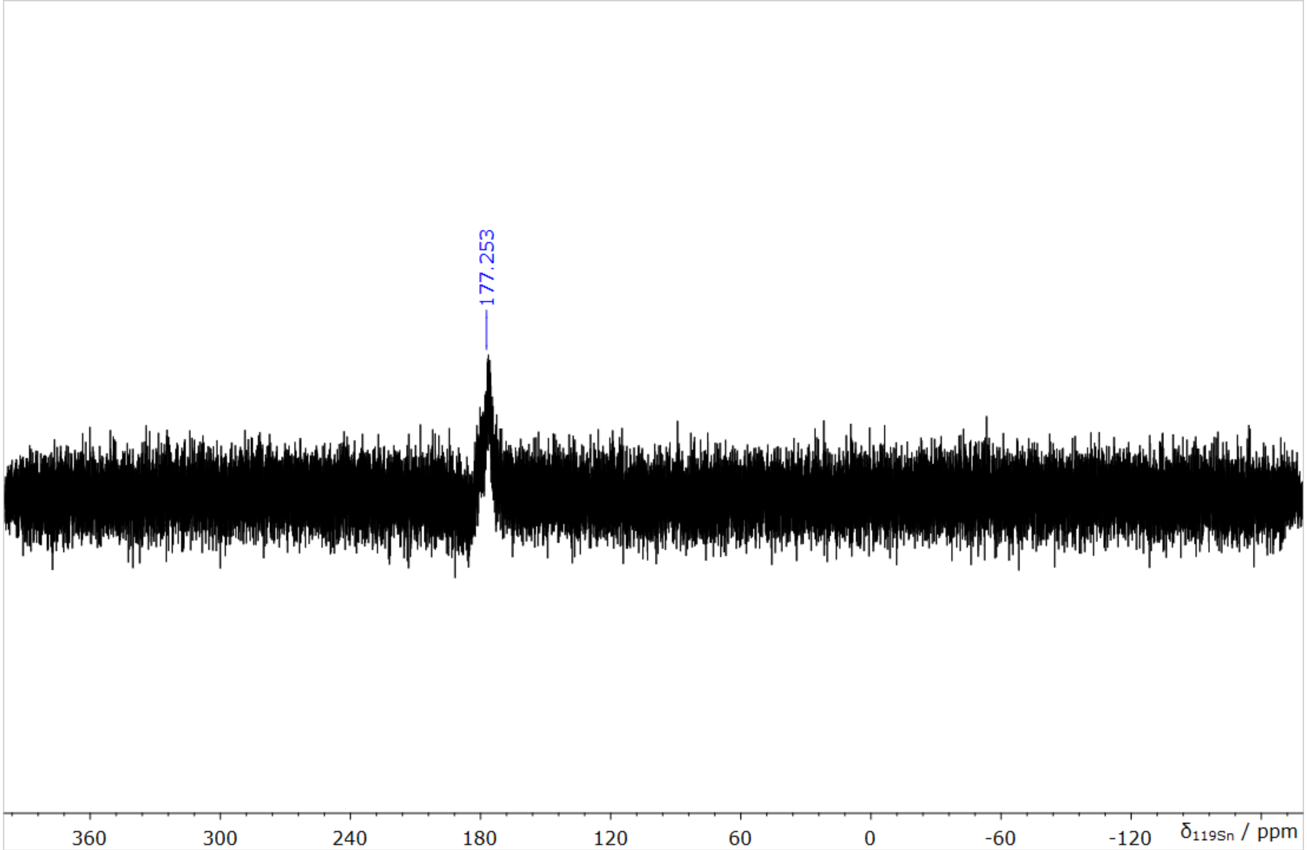

Figure S3. ^{119}Sn NMR spectrum of Bis(ferrocenyl)tetrathiastannolane **4a** in C_6D_6.

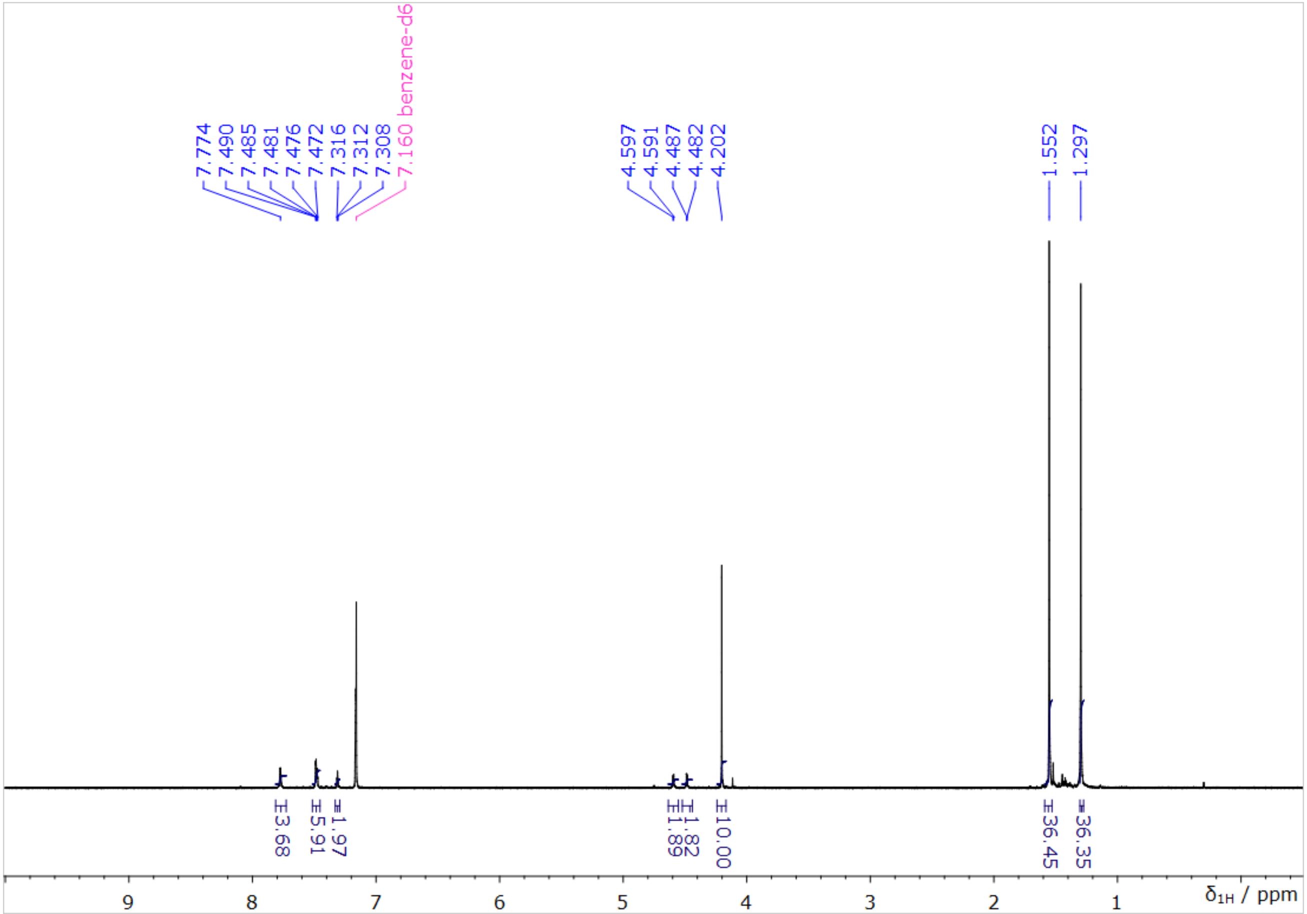

Figure S4. ^{1}H NMR spectrum of Bis(ferrocenyl)tetraselenastannolane **6** in C_6D_6.

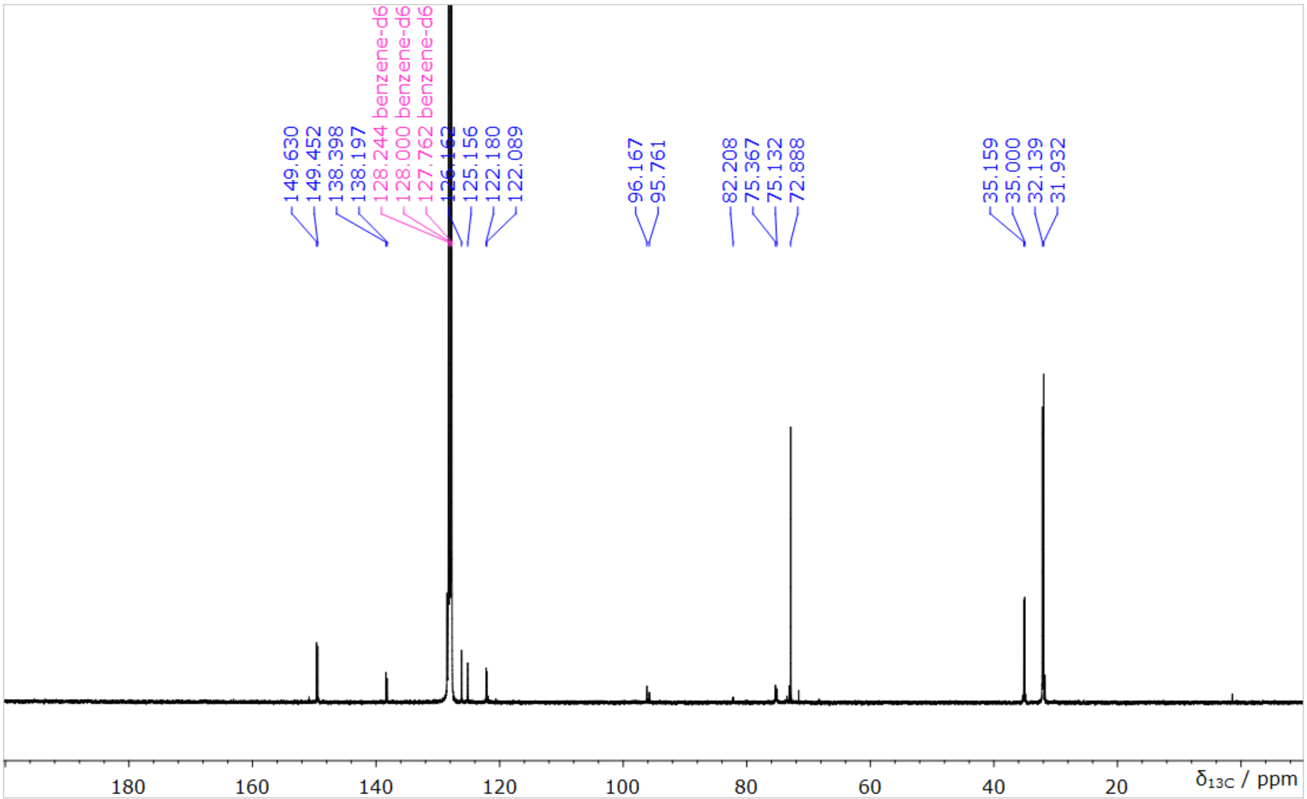

Figure S5. $^{13}C\{^1H\}$ NMR spectrum of Bis(ferrocenyl)tetraselenastannolane **6** in C_6D_6.

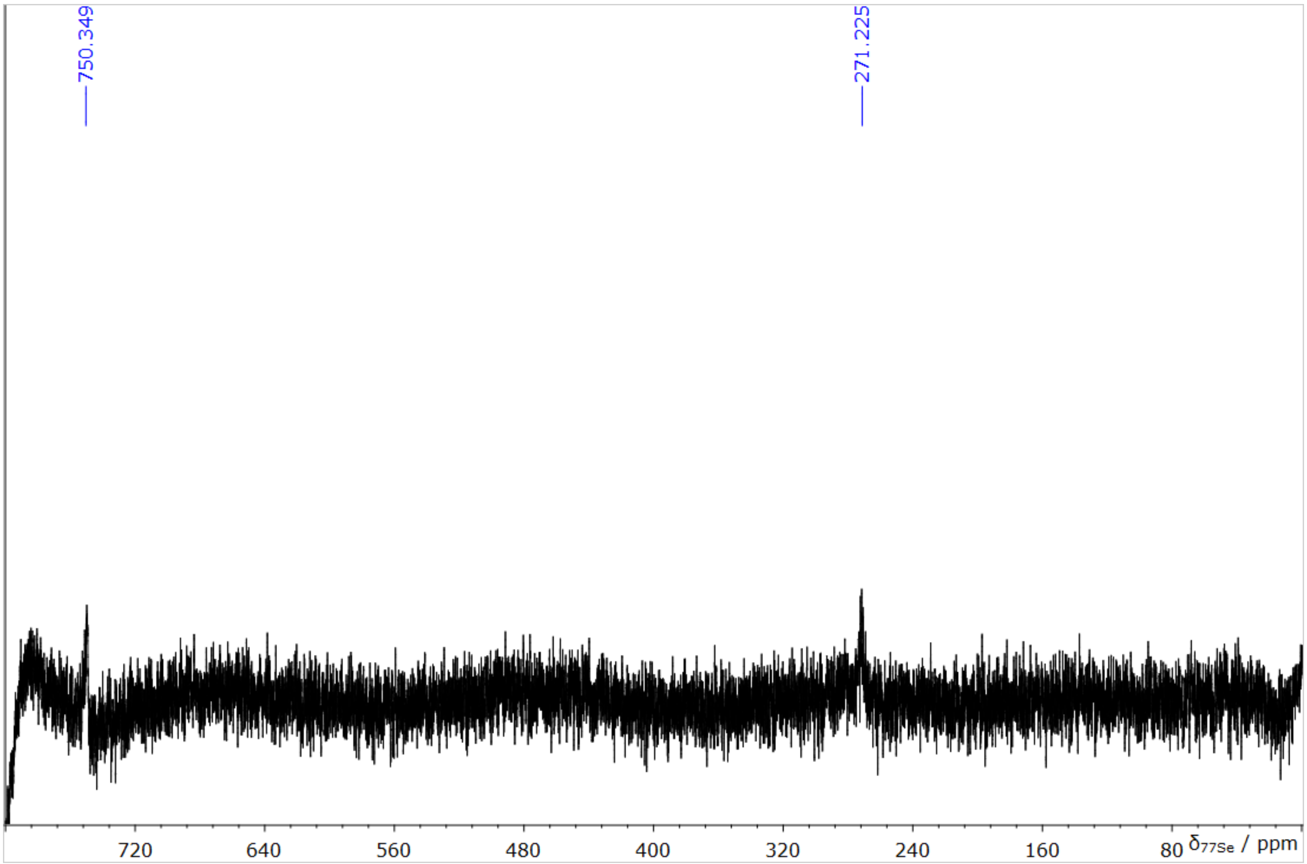

Figure S6. ^{77}Se NMR spectrum (C_6D_6, 76 MHz) of Bis(ferrocenyl)tetraselenastannolane **6**.

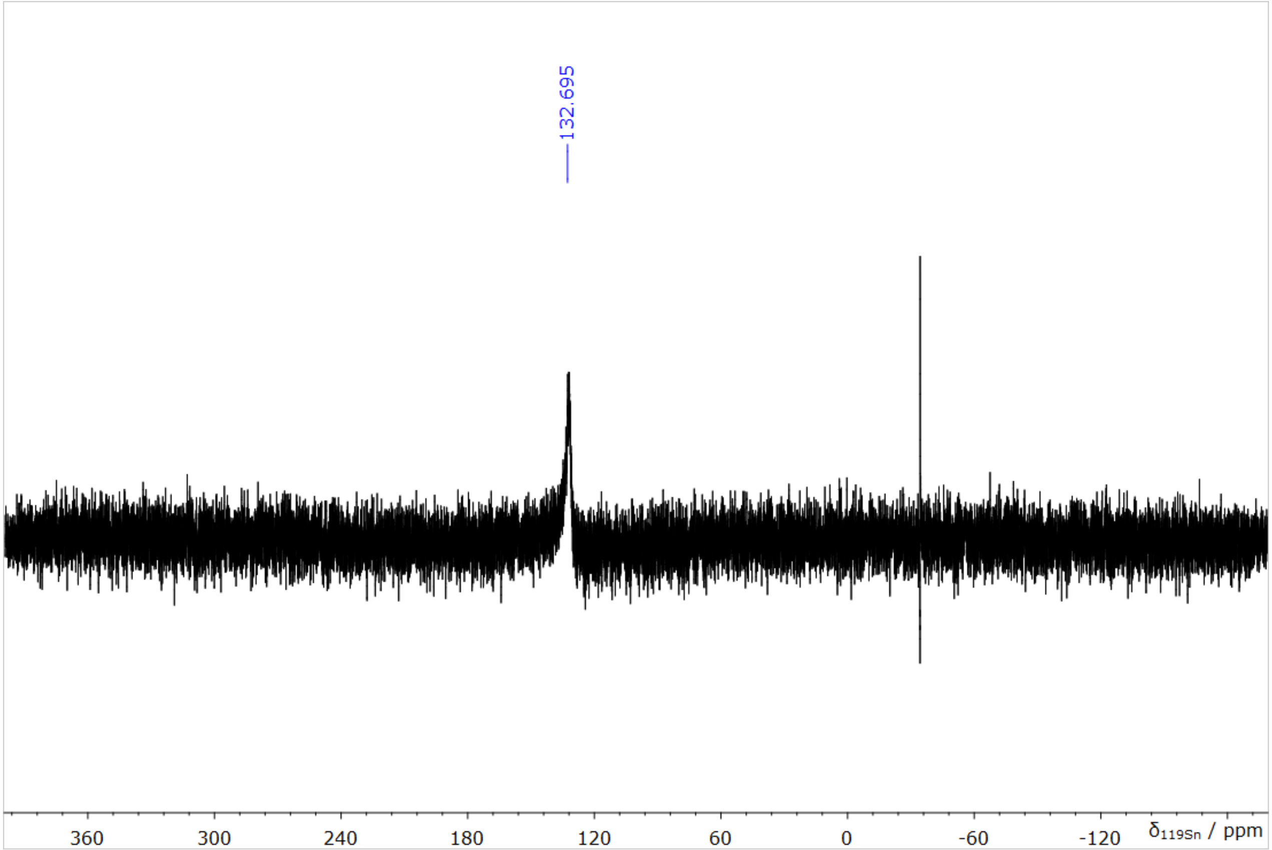

Figure S7. $^{119}Sn\{^{1}H\}$ NMR spectrum of Bis(ferrocenyl)tetraselenastannolane **6** in C_6D_6.

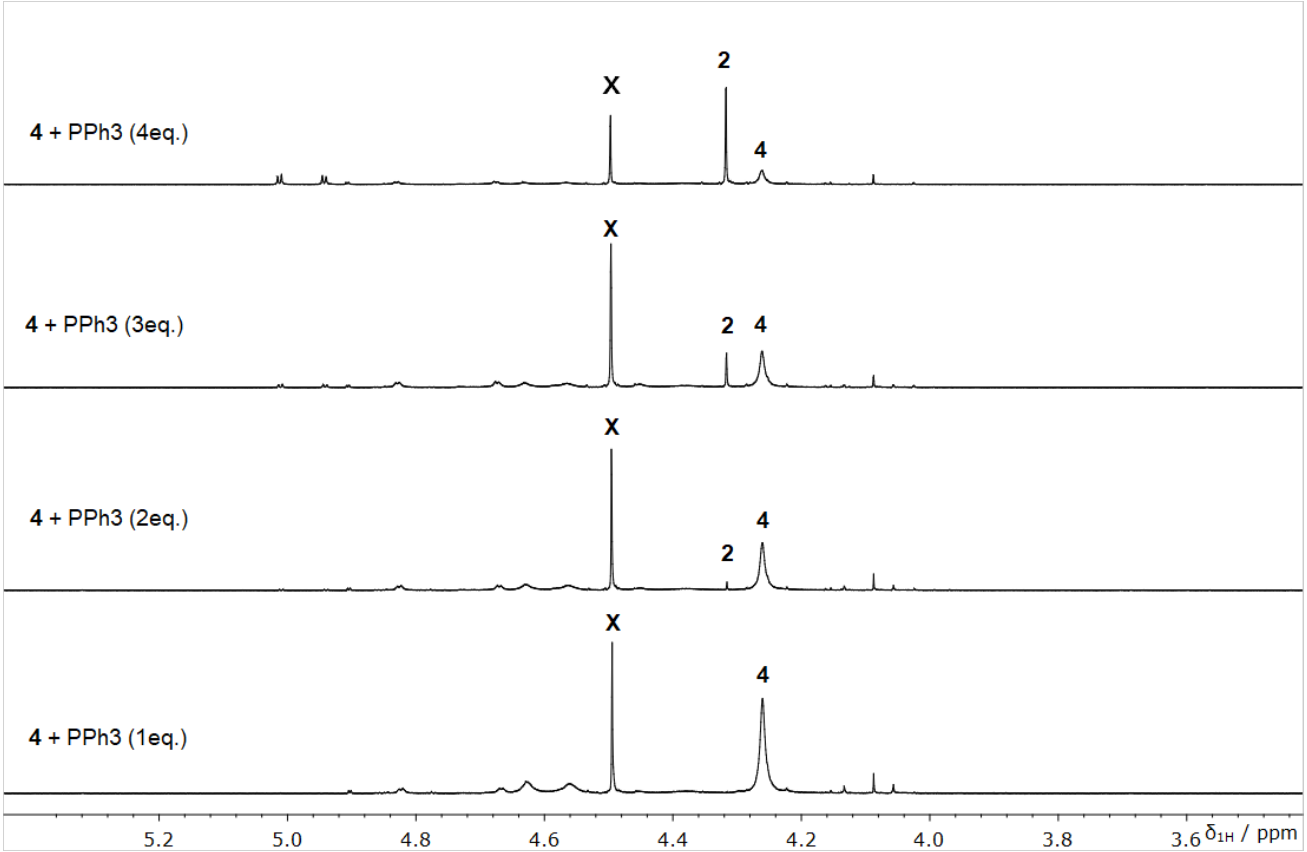

Figure S8. ^{1}H NMR spectra of the reaction mixtures of tetrathiastannolane **4** with triphenylphosphine.

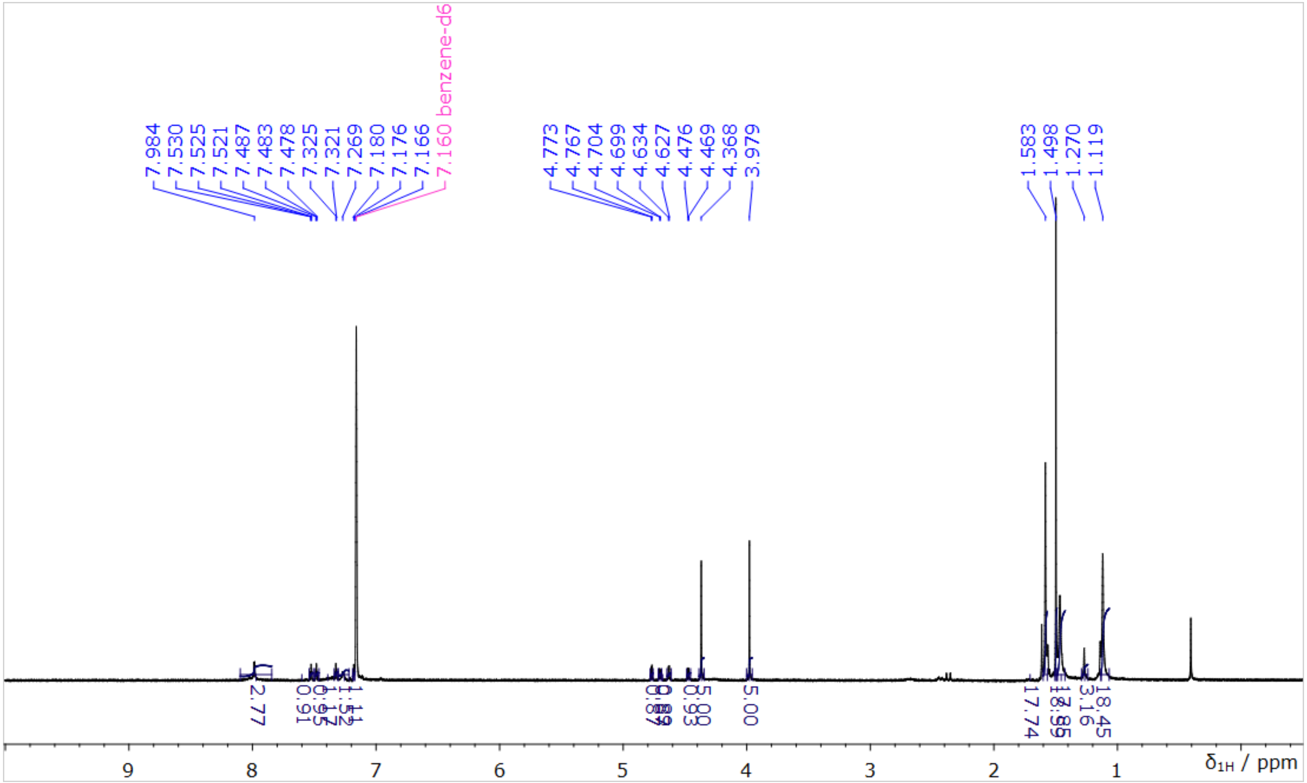

Figure S9. ^{1}H NMR spectrum of butadiene adduct **7** in C_6D_6.

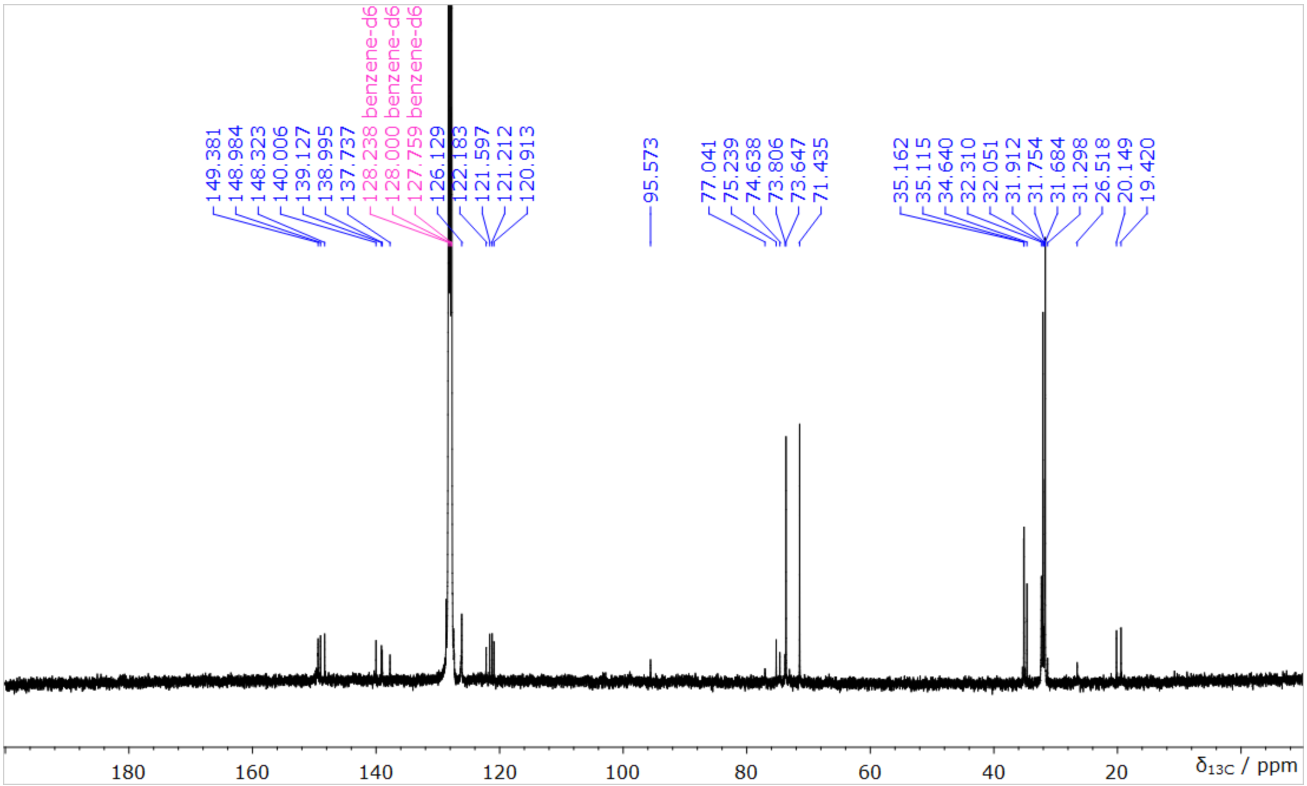

Figure S10. $^{13}C\{^{1}H\}$ NMR spectrum of butadiene adduct **7** in C_6D_6.

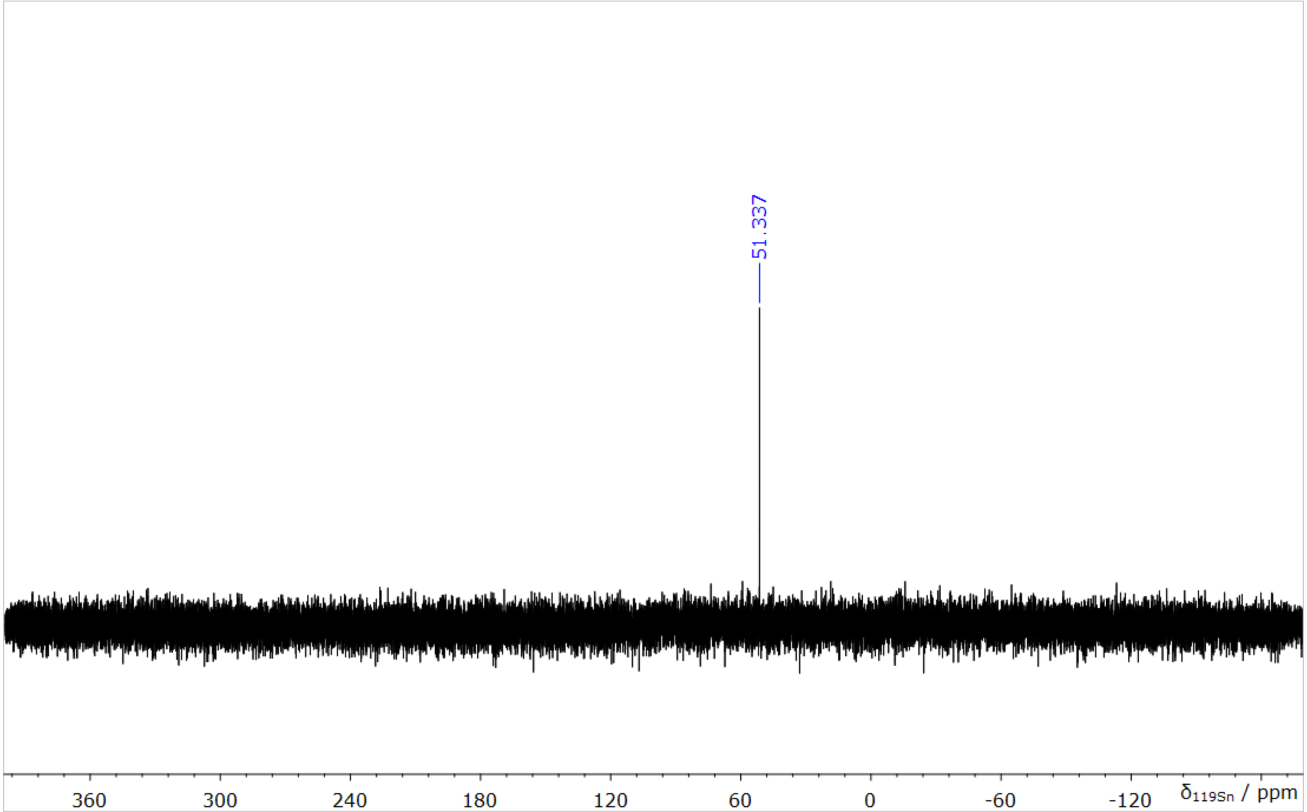

Figure S11. ^{119}Sn NMR spectrum of butadiene adduct **7** in C_6D_6.

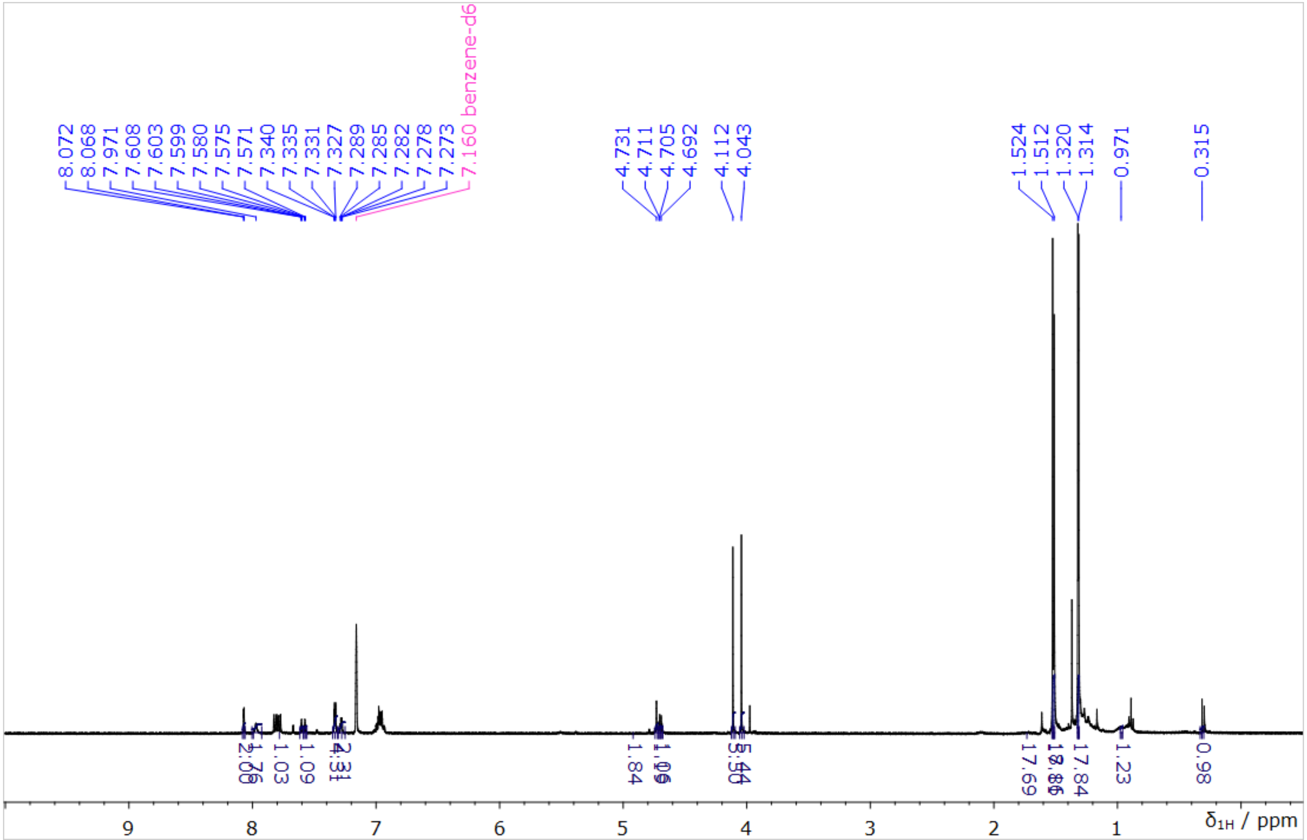

Figure S12. ^{1}H NMR spectrum of H_2O adduct **8** in C_6D_6.

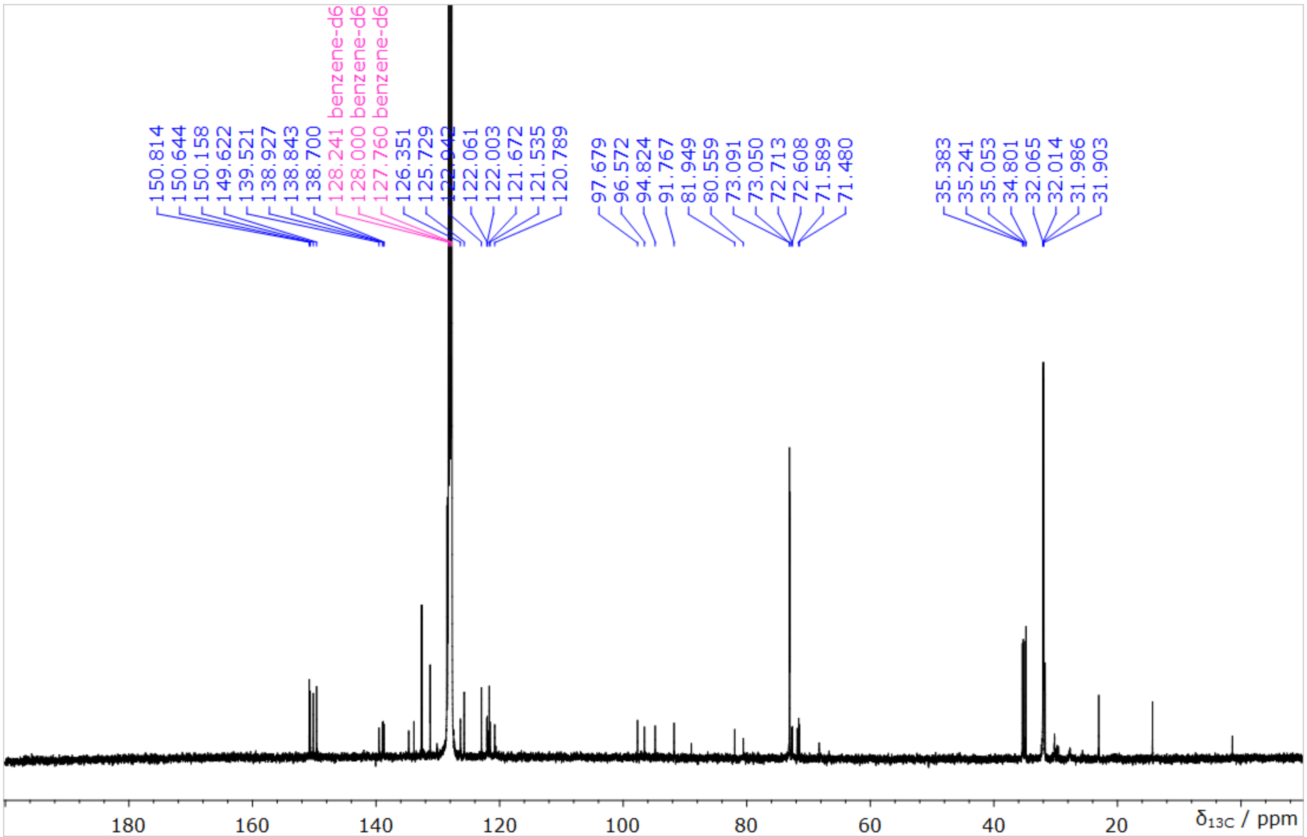

Figure S13. $^{13}C\{^{1}H\}$ NMR spectrum of H_2O adduct **8** in C_6D_6.

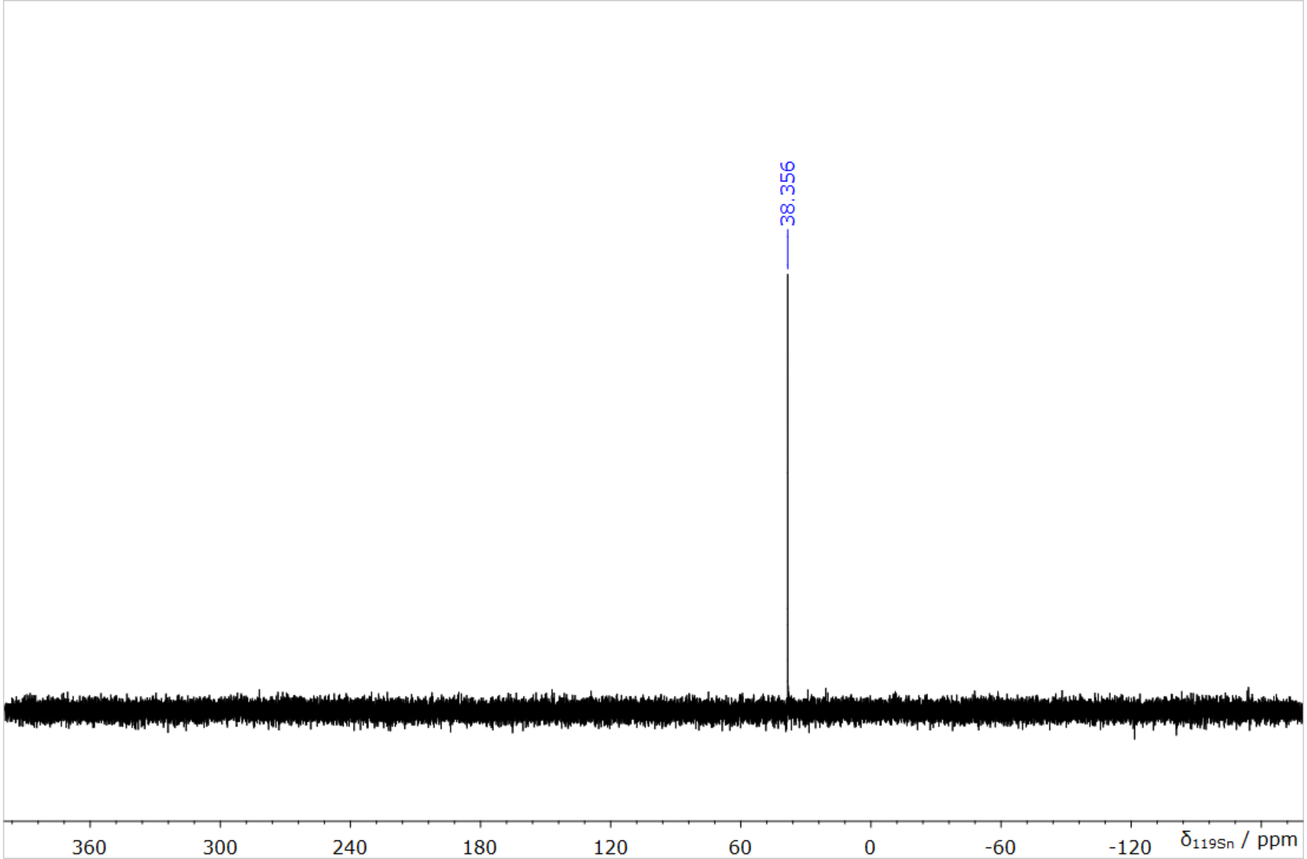

Figure S14. ^{119}Sn NMR spectrum of H_2O adduct **8** in C_6D_6.

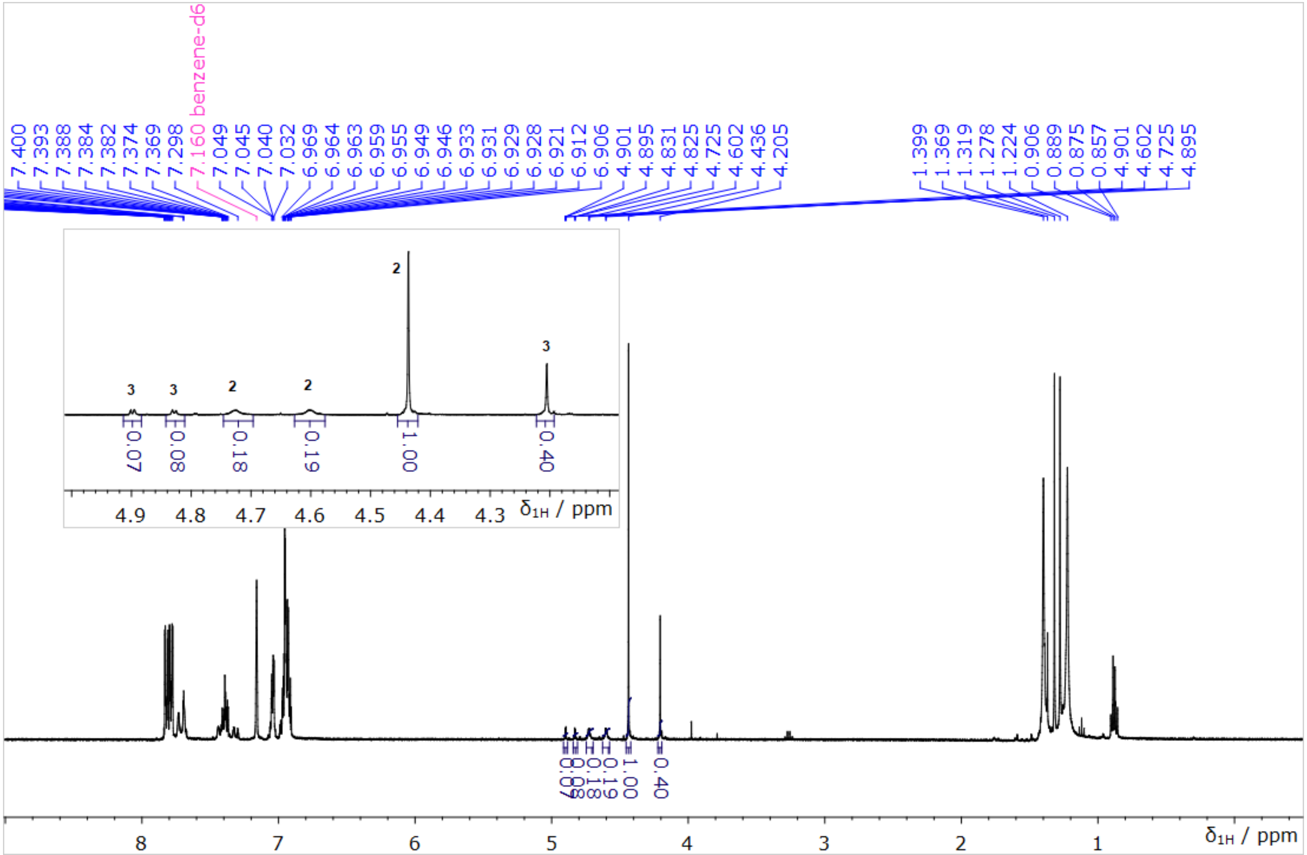

Figure S15. ^{1}H NMR spectrum of the reaction mixture of tetraselenastannolane **6** with triphenylphosphine.

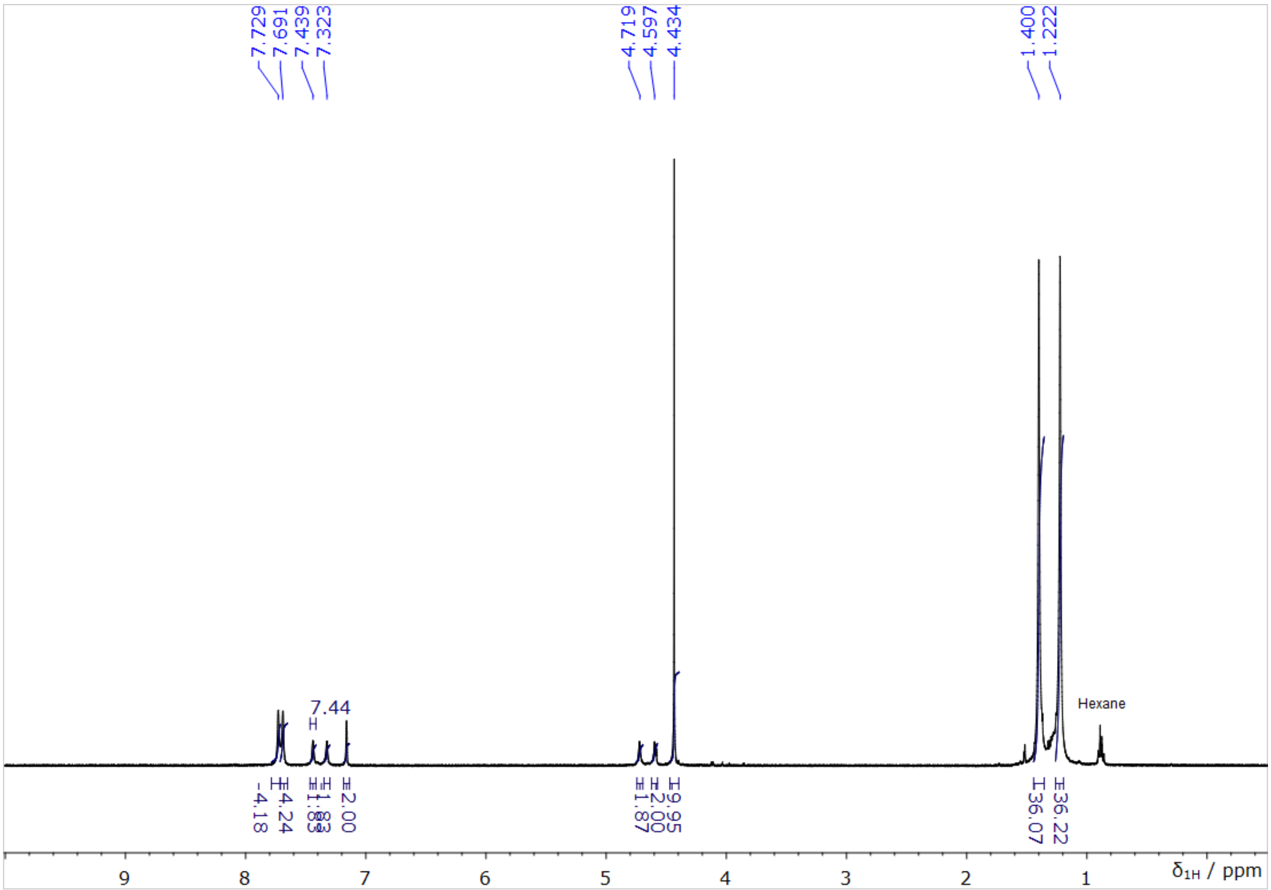

Figure S16. ^{1}H NMR spectrum of Bis(ferrocenyl)stannaneselone **2** in C_6D_6.

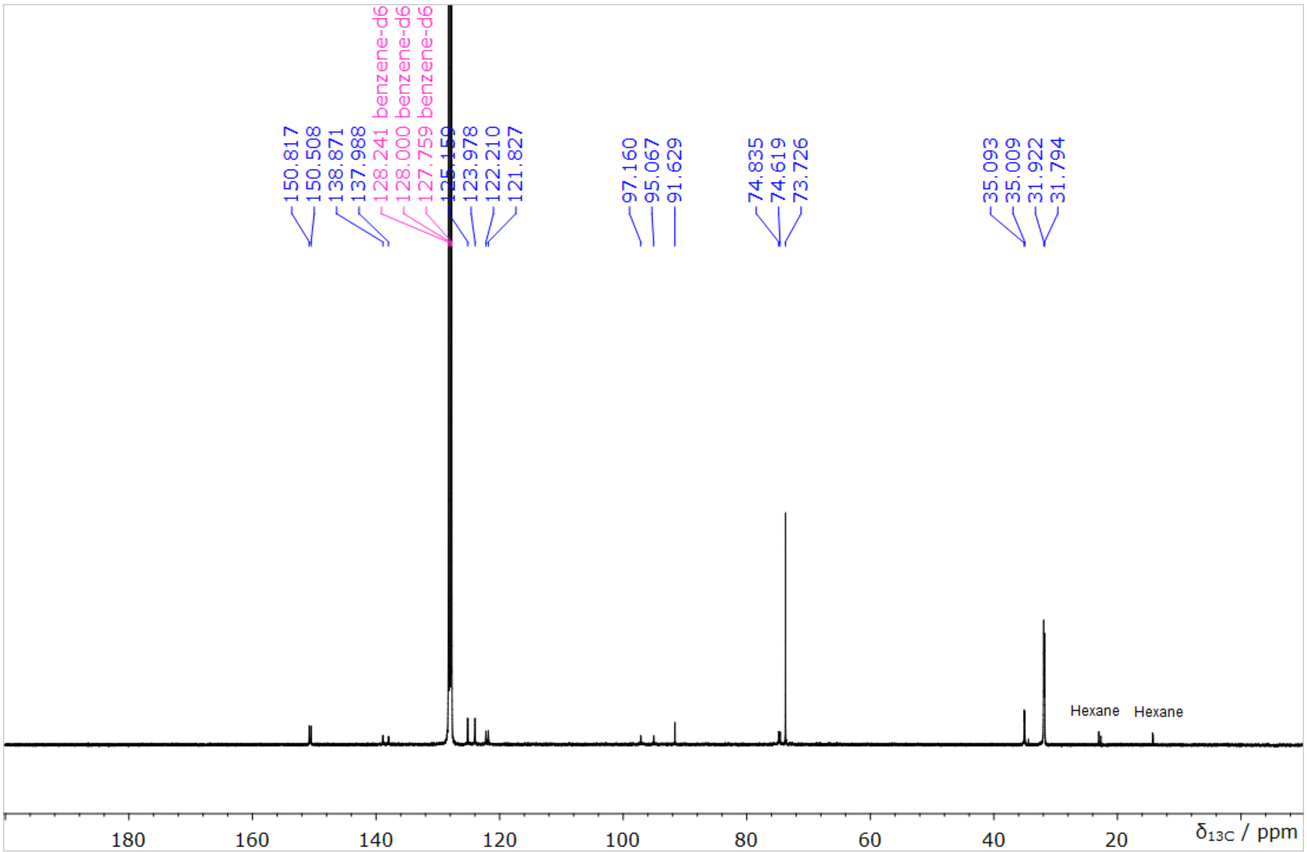

Figure S17. $^{13}C\{^{1}H\}$ NMR spectrum of Bis(ferrocenyl)stannaneselone **2** in C_6D_6.

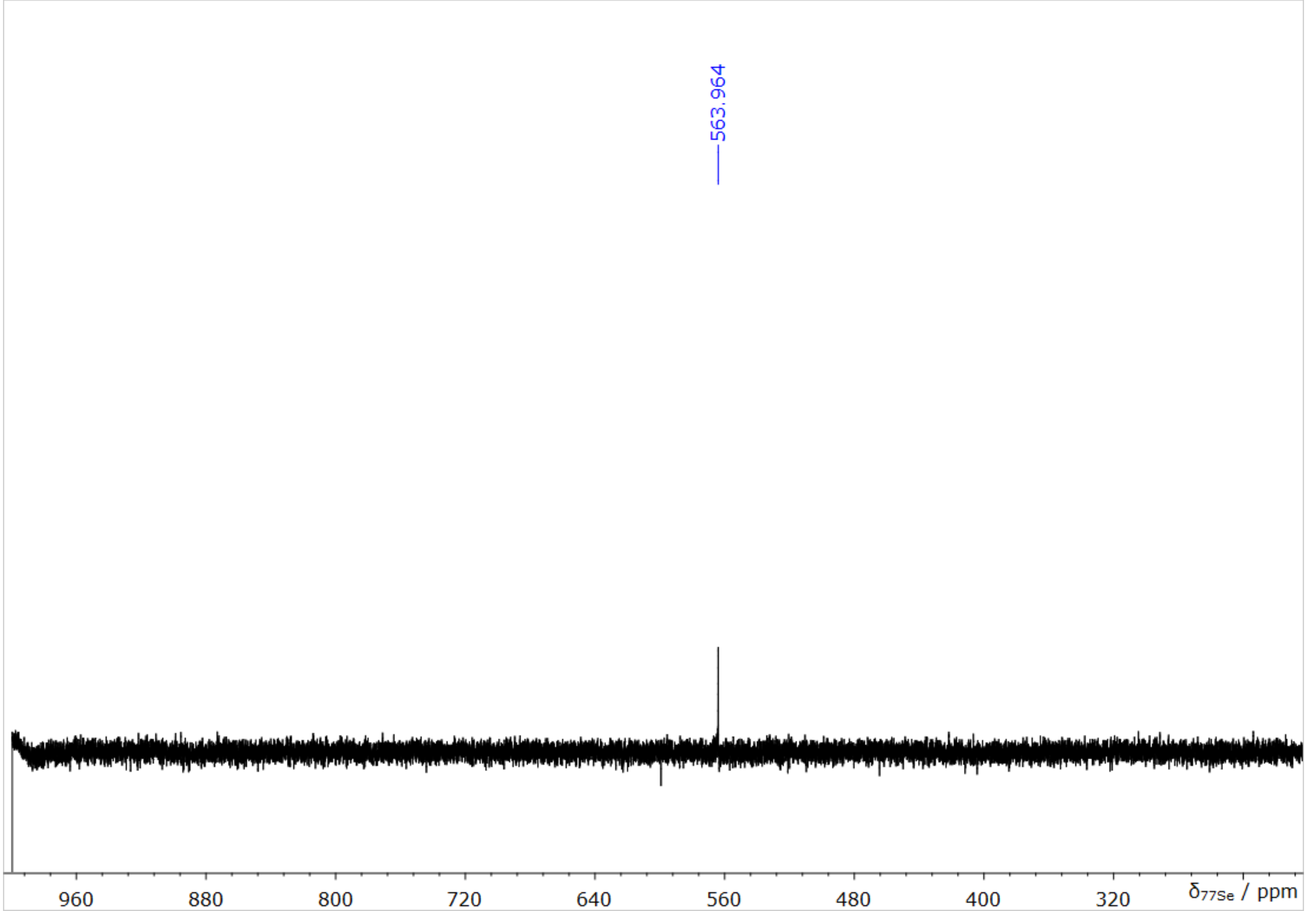

Figure S18. ^{77}Se NMR spectrum of Bis(ferrocenyl)stannaneselone **2** in C_6D_6

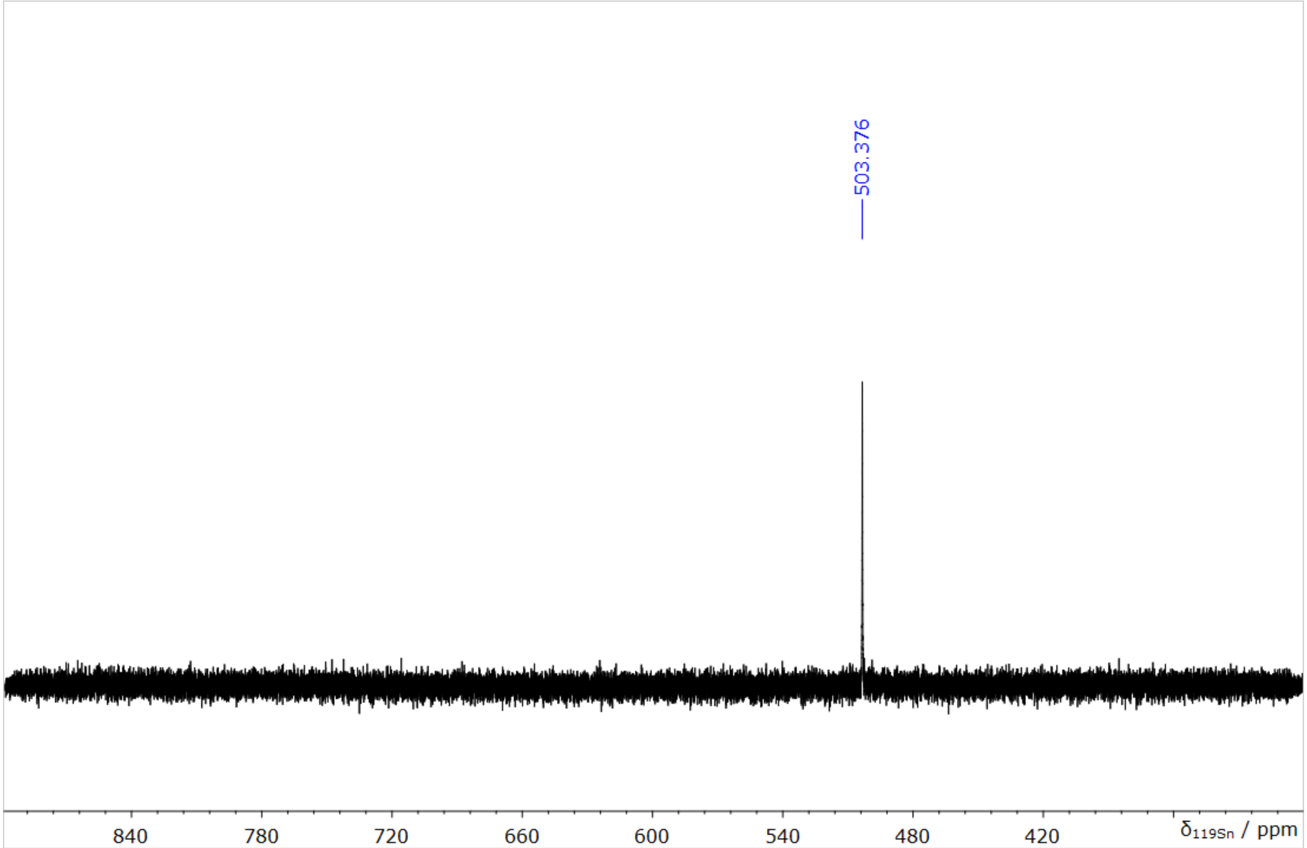

Figure S19. ^{119}Sn NMR spectrum of Bis(ferrocenyl)stannaneselone **2** in C_6D_6.

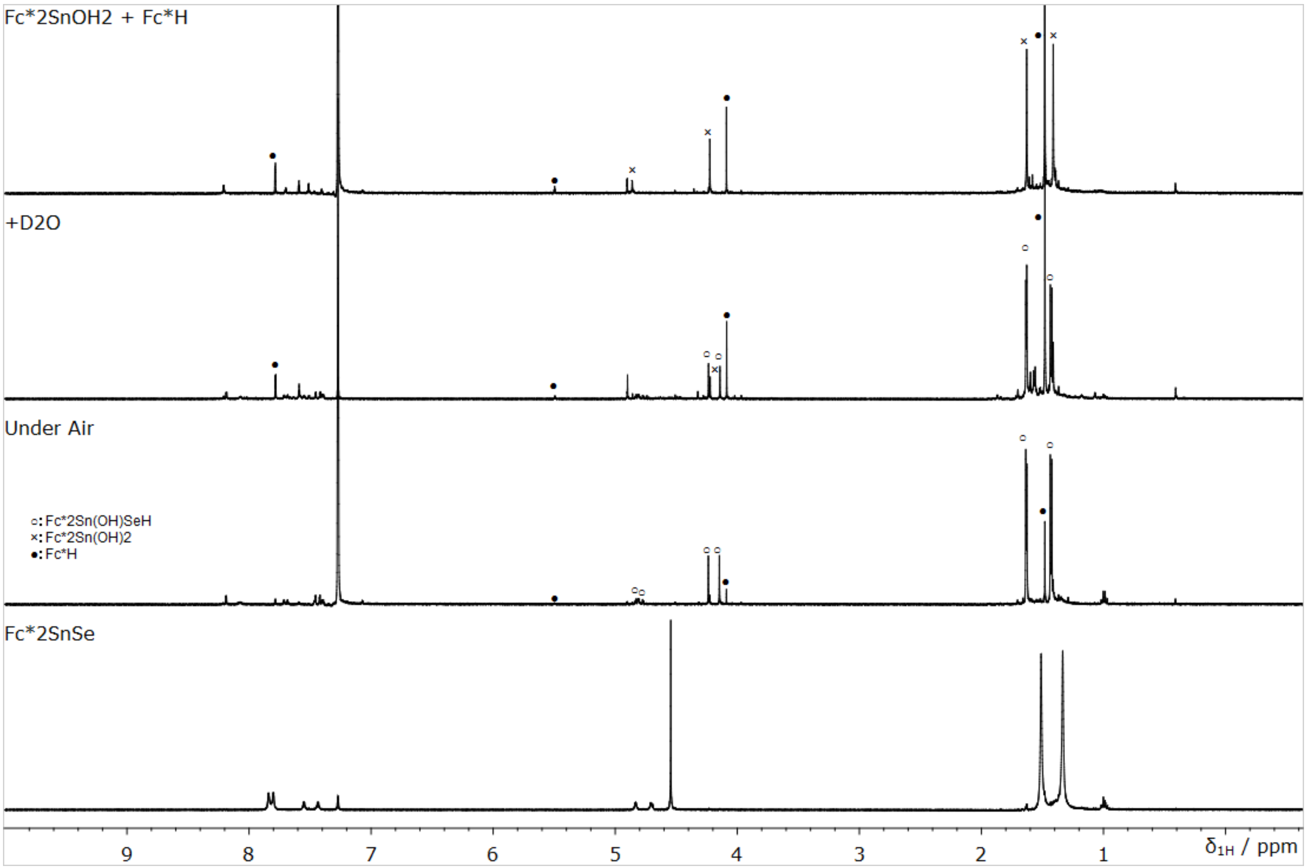

Figure S20. ^{1}H NMR spectra of hydrolyzed compounds from the reaction of **2** with H_2O.

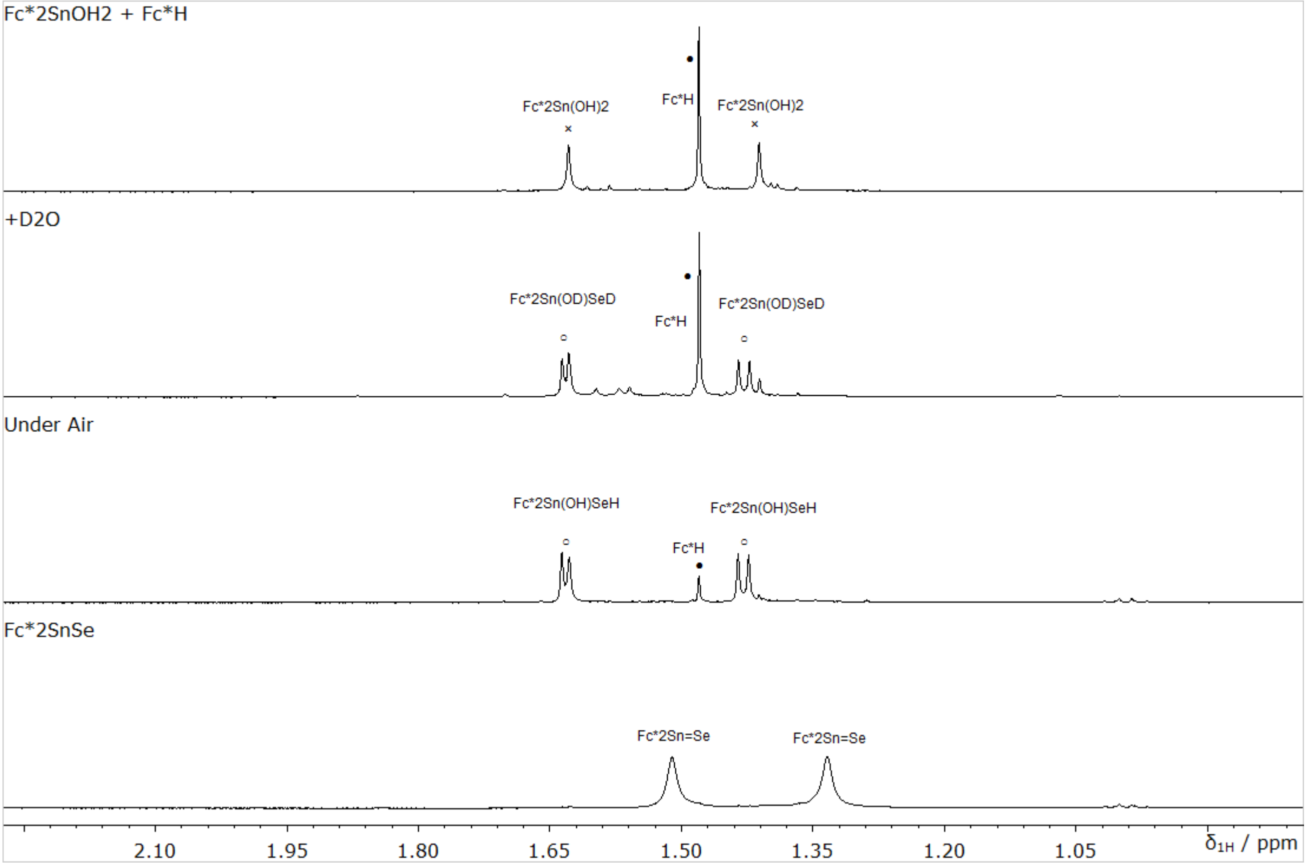

Figure S21. ^{1}H NMR spectra (around 1 ppm region) of the hydrolyzed compounds from the reaction of **2** with H_2O.

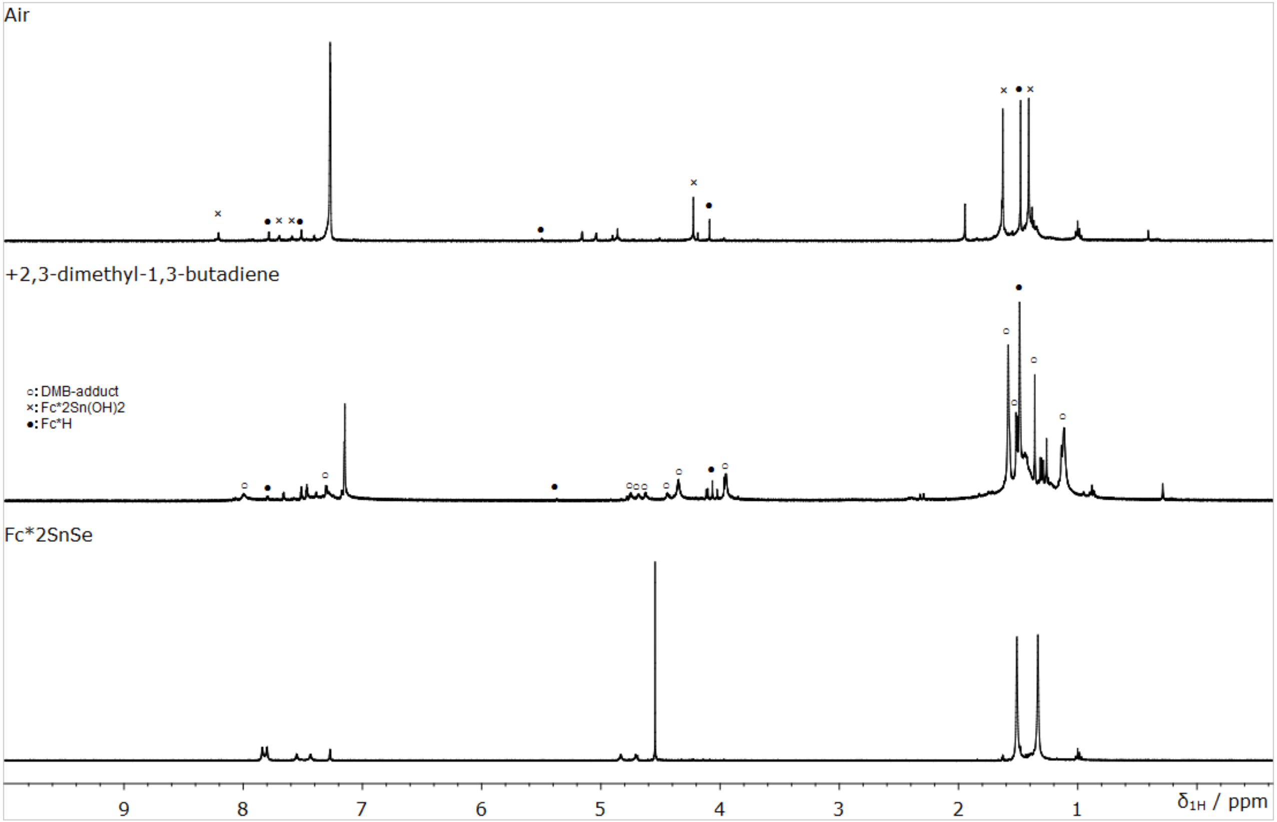

Figure S22. ^{1}H NMR spectra of **10** and its decomposition compounds from reaction of **2** with 2,3-dimethyl-1,3-butadiene.

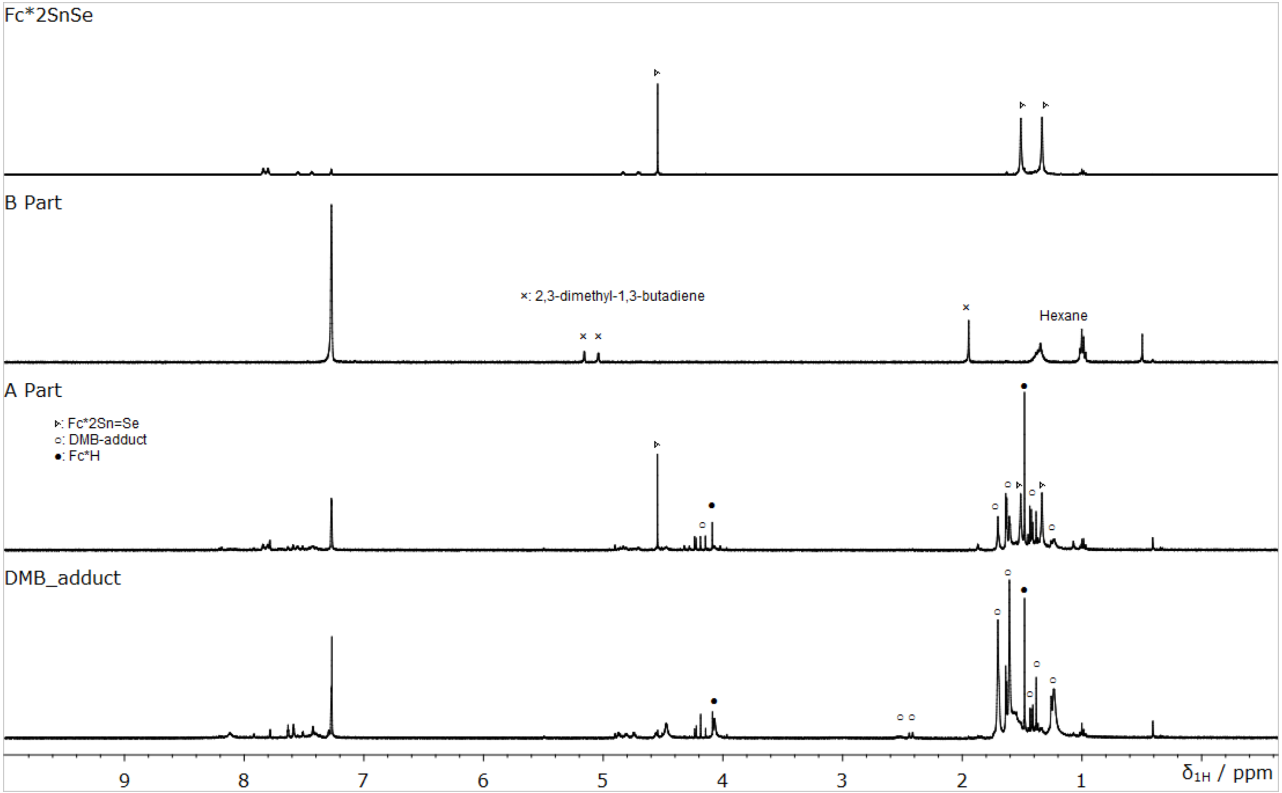

Figure S23. ^{1}H NMR spectra of DMB-adducts and re-generated DMB and **2** from [4 + 2] addition and retro [4+2] addition of **2** with 2,3-dimethyl-1,3-butadiene.

X-Ray Crystallographic Data

Table S1. Crystal data and data collection of **2·0.5(C_6H_6)**, **4a**, **4b**, **6**, **7**, and **8**.

Compound	**2·0.5(C_6H_6)**	**4a**	**4b**	**6**	**7**	**8**
Formula	$C_{79}H_{101}Fe_2SeSn$	$C_{76}H_{98}Fe_2S_4Sn$	$C_{76}H_{98}Fe_2S_{5.2}Sn$	$C_{76}H_{98}Fe_2Se_{4.2}Sn$	$C_{88}H_{114}Fe_2SSn$	$C_{82}H_{114}Fe_2OSSn$
Molecular Weight	2719.88	1370.17	1408.64	1573.56	1434.24	1378.18
Temperature	–170 °C	–170 °C	–170 °C	–170 °C	–170 °C	–170 °C
λ (Å)	0.71073	0.71073	0.71073	0.71073	0.71073	0.71073
Crystal size / mm^3	0.10×0.05×0.03	0.10×0.08×0.04	0.10×0.05×0.02	0.12×0.05×0.03	0.15×0.08×0.05	0.15×0.08×0.05
Crystal system	Monoclinic	Monoclinic	Orthorhombic	Monoclinic	Triclinic	Monoclinic
Space group	$P2_1/n$ (#14)	$P2_1/n$ (#14)	*Pnna* (#52)	$P2_1/n$ (#14)	*P*-1 (#2)	*P*2/*c* (#13)
a /Å	19.3954(3)	15.4833(2)	18.3966(2)	25.7657(3)	12.6041(1)	11.5071(1)
b /Å	35.2095(5)	22.5112(2)	30.5224(4)	11.3819(1)	13.7615(1)	16.2370(2)
c /Å	21.6237(3)	20.9383(3)	13.0978(2)	25.9495(3)	23.5222(2)	20.2036(2)
α /deg	90	90	90	90	97.278(1)	90
β / deg	113.369(2)	108.0180(10)	90	111.185(1)	103.250(1)	104.665(1)
γ / deg	90	90	90	90	100.371(1)	90
V /Å^3	13555.5(4)	6940.10(15)	7354.53(17)	7095.72(14)	3845.90(6)	3651.88(7)
Z	8	4	4	4	2	2
μ / mm^{-1}	1.368	0.931	0.913	2.948	0.765	0.803
$D_{calcd.}$ / g·cm^{-3}	1.333	1.311	1.272	1.473	1.239	1.253
θ_{max}	27.485	28.660	28.696	26.000	29.141	26.999
Collected Refl.	225838	113062	121462	199309	66600	114581
Refl./restr./param.	31060/144/1600	17073/0/772	9273/156/447	13930/84/913	19331/0/905	7974/216/541
Completeness	99.9	99.9	99.9	100	99.9	99.9
GOF	1.026	1.069	1.237	1.139	1.046	1.068
R_1 (I>2σ(I))	0.0375	0.0333	0.0449	0.0480	0.0363	0.0315
wR_2 (I>2σ(I))	0.0915	0.0742	0.0924	0.1107	0.0930	0.0764
R_1 (all data)	0.0470	0.0388	0.0513	0.0529	0.0383	0.0348
wR_2 (all data)	0.0962	0.0765	0.0943	0.1132	0.0943	0.0780
Largest diff. peak and hole /e·Å^{-3}	1.954, –1.662	1.933, –0.612	0.756, –0.583	2.682, –1.324	2.236, –0.671	0.594, −1.471
CCDC	2463980	2463981	2463982	2463983	2463984	2463985

·References

[S1] T. Sasamori, Y. Suzuki and N. Tokitoh, *Organometallics*, **2014**, *33*, 6696–6699.

[S2] K. Iijima, K. Sugamata, P. K. Majhi, T. Sasamori, *Eur. J. Inorg. Chem.* **2024**, *2024*, e202400198.

[S3] I. Uson and G. M. Sheldrick, *Acta Cryst. Sect. D.* **2018,** *74*, 106-116.

[S4] (a) G. M. Sheldrick, *Acta Cryst.,* 2015, A71, 3-8. (b) G. M. Sheldrick, *Acta Cryst.,* 2015, C71, 3-8.

[S5] Wakita, K. (2001). Yadokari-XG. Software for Crystal Structure Analyses. Release of Software (Yadokari-XG 2009) for Crystal Structure Analyses, Kabuto, C., Akine, S., Nemoto, T. and Kwon, E. *J. Cryst. Soc. Jpn.*, **2009,** *51*, 218-224.

[S6] Gaussian 16, Revision C.01, M. J. Frisch, G. W. Trucks, H. B. Schlegel, G. E. Scuseria, M. A. Robb, J. R. Cheeseman, G. Scalmani, V. Barone, G. A. Petersson, H. Nakatsuji, X. Li, M. Caricato, A. V. Marenich, J. Bloino, B. G. Janesko, R. Gomperts, B. Mennucci, H. P. Hratchian, J. V. Ortiz, A. F. Izmaylov, J. L. Sonnenberg, D. Williams-Young, F. Ding, F. Lipparini, F. Egidi, J. Goings, B. Peng, A. Petrone, T. Henderson, D. Ranasinghe, V. G. Zakrzewski, J. Gao, N. Rega, G. Zheng, W. Liang, M. Hada, M. Ehara, K. Toyota, R. Fukuda, J. Hasegawa, M. Ishida, T. Nakajima, Y. Honda, O. Kitao, H. Nakai, T. Vreven, K. Throssell, J. A. Montgomery, Jr., J. E. Peralta, F. Ogliaro, M. J. Bearpark, J. J. Heyd, E. N. Brothers, K. N. Kudin, V. N. Staroverov, T. A. Keith, R. Kobayashi, J. Normand, K. Raghavachari, A. P. Rendell, J. C. Burant, S. S. Iyengar, J. Tomasi, M. Cossi, J. M. Millam, M. Klene, C. Adamo, R. Cammi, J. W. Ochterski, R. L. Martin, K. Morokuma, O. Farkas, J. B. Foresman, and D. J. Fox, Gaussian, Inc., Wallingford CT, **2016**.
